# *k*-mer Similarity, Networks of Microbial Genomes, and Taxonomic Rank

**DOI:** 10.1128/mSystems.00257-18

**Published:** 2018-11-20

**Authors:** Guillaume Bernard, Paul Greenfield, Mark A. Ragan, Cheong Xin Chan

**Affiliations:** a Institute for Molecular Bioscience, The University of Queensland, Brisbane, QLD, Australia; b Commonwealth Scientific and Industrial Research Organisation (CSIRO), North Ryde, NSW, Australia; c School of Chemistry and Molecular Biosciences, The University of Queensland, Brisbane, QLD, Australia; University College Cork

**Keywords:** core functions, *k*-mers, networks, phylogenetic analysis, phylogenomics

## Abstract

Microbial genomes have been shaped by parent-to-offspring (vertical) descent and lateral genetic transfer. These processes can be distinguished by alignment-based inference and comparison of phylogenetic trees for individual gene families, but this approach is not scalable to whole-genome sequences, and a tree-like structure does not adequately capture how these processes impact microbial physiology. Here we adopted alignment-free approaches based on *k*-mer statistics to infer phylogenomic networks involving 2,783 completely sequenced bacterial and archaeal genomes and compared the contributions of rRNA, protein-coding, and plasmid sequences to these networks. Our results show that the phylogenomic signal arising from ribosomal RNAs is strong and extends broadly across all taxa, whereas that from plasmids is strong but restricted to closely related groups, particularly *Proteobacteria*. However, the signal from the other chromosomal regions is restricted in breadth. We show that mean *k*-mer similarity can correlate with taxonomic rank. We also link the implicated *k*-mers to genome annotation (thus, functions) and define core *k*-mers (thus, core functions) in specific phyletic groups. Highly conserved functions in most phyla include amino acid metabolism and transport as well as energy production and conversion. Intracellular trafficking and secretion are the most prominent core functions among *Spirochaetes*, whereas energy production and conversion are not highly conserved among the largely parasitic or commensal *Tenericutes*. These observations suggest that differential conservation of functions relates to niche specialization and evolutionary diversification of microbes. Our results demonstrate that *k*-mer approaches can be used to efficiently identify phylogenomic signals and conserved core functions at the multigenome scale.

**IMPORTANCE** Genome evolution of microbes involves parent-to-offspring descent, and lateral genetic transfer that convolutes the phylogenomic signal. This study investigated phylogenomic signals among thousands of microbial genomes based on short subsequences without using multiple-sequence alignment. The signal from ribosomal RNAs is strong across all taxa, and the signal of plasmids is strong only in closely related groups, particularly *Proteobacteria*. However, the signal from other chromosomal regions (∼99% of the genomes) is remarkably restricted in breadth. The similarity of subsequences is found to correlate with taxonomic rank and informs on conserved and differential core functions relative to niche specialization and evolutionary diversification of microbes. These results provide a comprehensive, alignment-free view of microbial genome evolution as a network, beyond a tree-like structure.

## Abstract (precis)

Genome evolution of microbes involves parent-to-offspring descent, and lateral genetic transfer that convolutes the phylogenomic signal. This study investigated phylogenomic signals among thousands of microbial genomes based on short subsequences without using multiple-sequence alignment. The signal from ribosomal RNAs is strong across all taxa, and the signal of plasmids is strong only in closely related groups, particularly *Proteobacteria*. However, the signal from other chromosomal regions (∼99% of the genomes) is remarkably restricted in breadth. The similarity of subsequences is found to correlate with taxonomic rank and informs on conserved and differential core functions relative to niche specialization and evolutionary diversification of microbes. These results provide a comprehensive, alignment-free view of microbial genome evolution as a network, beyond a tree-like structure.

## INTRODUCTION

For nearly 100 years following the discovery of diverse bacteria by Pasteur, Koch, Cohn, and others in the latter decades of the 19th century (1), little was known of how these organisms might be related among themselves or to the rest of the living world. This began to change with the recognition that ribosomal RNAs are present in all living cells and contain structural domains that, by virtue of their differential entanglements with core molecular functions and their interactions with greater or lesser numbers of other components of the translational apparatus, can inform on evolutionary history across a range of temporal scales “much as the hands of a clock separately indicate hours, minutes, and seconds” (2). Given the central role of translation in the emergence of phenotype from genotype and the number and interrelatedness of these structural and functional constrains, it was assumed that statistical analysis of rRNA sequences would recover the tree of vertical descent not merely of the corresponding genes but also, much more interestingly, of the host organisms. As it happened, the PCR method was invented at about the same time (3), and the presence of conserved 5′ and 3′ regions made the rRNA gene an attractive target for amplification and sequencing. Thus, Darwin’s Great Tree of Life quickly became universal, and, as a bonus, *Archaebacteria* (*Archaea*) were recognized as a distinct domain of living organisms.

As molecular evolutionary studies were extended into families of protein-coding genes, congruent topologies were often (but not always) recovered (4). In contrast to expectation, instances of incongruence often failed to be resolved as data sets grew larger and statistical methodology for phylogenetic inference improved. It also became clear that many microbes can exchange genetic material through the mediation of plasmids or phage and/or take up DNA from their environment. Depending on the breadth and granularity of the data, phylogenetic trees inferred for regions of lateral origin may thus contain edges that directly connect lineages that are nonadjacent in the rRNA tree. That is, lateral genetic transfer creates phylogenomic networks. Plasmid and phage sequences in particular are expected to increase the connectivity of phylogenomic networks, although any genetic material that becomes established in a new host genome after transmission by such a vector can contribute.

The resulting pattern of phylogenomic relationships has been described (by the use of diverse metaphors) as fundamentally treelike (5, 6), as a tree overgrown with tiny vines (7), as a ring (8, 9), as a coral (10), as a web (7, 11), as a network with some treelike regions (12), or simply as a network (13, 14). Networks of lateral genetic transfer (11, 13) highlighted the need to visualize contributions of different genomic regions on a broad scale.

Complete genome sequences are now available for thousands of bacterial and archaeal species, making it possible to assess microbial evolution globally and, often, at considerable phyletic depth. However, until recently these studies were necessarily biased in favor of alignable regions, i.e., genes, as classical phylogenomic workflows are based on multiple-sequence alignment (MSA) of putative orthogroups. Recently, so-called alignment-free (AF) approaches have been shown to perform well in phylogenetic inference from simulated and empirical (microbial genome) data sets (15; see references 16, 17, and 18 for recent reviews).

An important class of AF methods consists of approaches based on subsequences of fixed length, known as *k*-mers. These methods typically compute a matrix of distances on the basis of, e.g., the number of shared *k*-mers, which can then be used to generate a tree by the use of, e.g., neighbor joining (19) or a similarity network (20). Alternatively, *k*-mers of lateral origin can be recognized (11, 21, 22) and used to generate a directional network in which the edges natively represent inferred lateral relationships. The use of *k*-mers in phylogenetics is biologically intuitive (23, 24); the earlier works of Carl Woese and colleagues (25–27) showed that short (enzymatically digested) oligonucleotides of 16S/18S ribosomal RNAs carry significant phylogenetic (and thus, homology) signal and reveal the three domains of life. AF approaches can recover homology signal among molecular sequences at the genome scale and have been successfully applied to genomes of bacteria and archaea (15, 28–30), organelles (31), plants (31), and primates (30) as well as to microbial metagenomes (30).

AF methods can be more robust than MSA-based approaches to among-site rate heterogeneity, compositional bias, rearrangement, and insertion-deletion events (15, 32) and are scalable for very large data sets (32, 33). We earlier generated an AF phylogenetic network for 143 bacterial and archaeal genomes (29) using pairwise *k*-mer distances computed using the *D*_2_^*S*^ statistic (34, 35). By varying similarity thresholds, we could easily display changes of network structure, e.g., the progressive separation of genomic lineages (29) or the disappearance of cliques (putative “genetic exchange communities” [11, 36]).

Here we used *k*-mer methods and the *D*_2_^*S*^ statistic to infer phylogenomic networks for 2,783 complete prokaryote genomes and investigated the contribution of different components of the data to the phylogenetic signals captured by AF methods. Specifically, we compared AF networks inferred using (i) complete genomic data sets, including plasmids, if any; (ii) chromosomal sequences without rRNA genes; (iii) only rRNA genes; and (iv) only plasmid sequences. Using an advanced database approach, we investigated the core functions that are specific to particular phyletic groups or genera on the basis of the shared *k*-mers.

## RESULTS

For each subset of the data (see above), we first calculated a distance *d* between the genomes in a given pair (*a* and *b*) using the *D*_2_^*S*^ distance measure and *k *=* *25 (see Materials and Methods). The value of *k *=* *25 was found to capture an adequate level of uniqueness among 1,121 complete bacterial genome sequences and is thus suitable for deriving a metric of relatedness among bacterial genomes (37). We transformed the distance between genome *a* and genome *b* (*d_ab_*) into a similarity value (*S_ab_*) and generated a similarity network using a method that we described previously (29). These networks capture the relatedness among these genomes, i.e., are phylogenomic, although the relative contributions of the vertical and lateral components (which may be admixed) depend on the subset of data used as input. Here we define a threshold *t* for which only edges with *S* values that are ≥*t* are considered in the network. To compare our results at the genome and phylum levels, we generated *I*-networks in which nodes represent distinct genome isolates and edges indicate evidence of shared *k*-mers and also generated *P*-networks in which nodes represent distinct phyla and edges represent the number of isolates (summed over both nodes) that share *k*-mers with isolates of the other phylum (see Materials and Methods). Given the taxon richness of *Proteobacteria*, we evaluated its subgroups (e.g., *Alphaproteobacteria* and *Betaproteobacteria*) as individual phyla. We then compared the *k*-mer networks based on the topological differences between them at different *t* values. All *I*- and *P*-networks of these 2,705 genome isolates are available at https://doi.org/10.14264/uql.2017.436.

### AF networks of microbial evolution.

**FIG 1:**
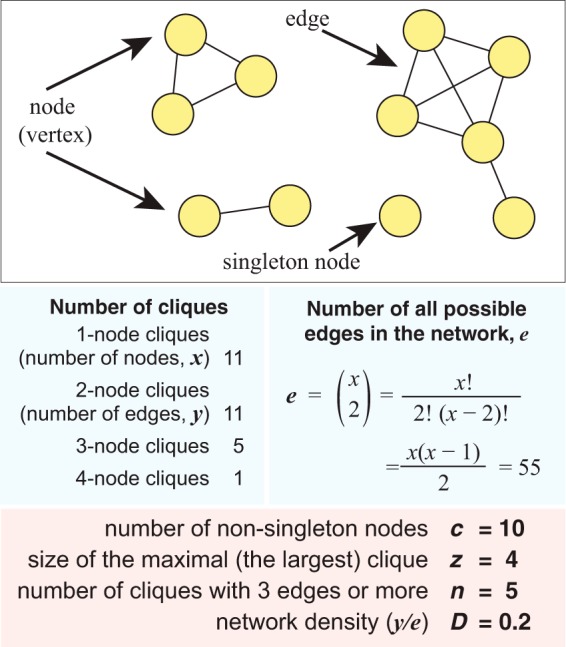
Definition of key terms of network characteristics used in this study. The example 11-node network is shown at the top, and the definition of each key term associated with this example network is shown at the bottom.

We first inferred phylogenomic networks based on a data set of 2,783 completely sequenced microbial genomes (2,618 bacterial genomes and 165 archaeal genomes [total of 9,582,718,896 bases]) downloaded from NCBI on 31 January 2016 (see Data Set S1 in the supplemental material), including plasmid sequences if present. Where two or more genomes had identical contents of 25-mers (*D*_2_^*S*^ distance = 0), only one was retained. We also removed edges for which the *D*_2_^*S*^ distance was >10; these genomes share ≤0.01% of 25-mers with any other genome. Following this filtering step, we took 2,705 genomes forward into subsequent analyses. For each network, we systematically assessed the number of nonsingleton nodes (*c*) (i.e., the number of nodes with one or more edges), the size of the maximal clique (i.e., the clique with the largest number of genomes) (*z*), and the number of cliques (*n*) across various levels of the similarity score threshold (*t*). We required a clique to contain three or more edges and defined *D* as the density of a network, i.e., the proportion of edges among all possible edges in a network (Fig. 1; see also Materials and Methods).

10.1128/mSystems.00257-18.10 DATA SET S1 The 2,783 completely sequenced microbial genomes used in this study. For each genome data set, the data source, the total number of bases, and the total number of 25-mers are shown. Download Data Set S1, XLSX file, 0.2 MB. Copyright © 2018 Bernard et al. 2018 Bernard et al. This content is distributed under the terms of the Creative Commons Attribution 4.0 International license.

The network topology changes substantially with similarity threshold: at *t *=* *0, *c *=* *2,705 and *z *=* *2,704, compared to *c *=* *1,358 and *z *=* *48 at *t *=* *9 (Table 1). As we increase the stringency of the threshold of shared similarity, the network becomes less connected, and distinct subsets corresponding to diverse taxa (i.e., phyla, classes, and genera) start to emerge. In this network, many bacterial phyla are represented in a single subgraph at *t *=* *4, most phyla can be identified as distinct sets at *t *=* *5, and all proteobacterial classes are separate from each other at *t *>* *5.

**TABLE 1:** Characteristics of the phylogenomic network of 2,705 prokaryote genomes based on complete genomic data sets

Threshold	No. of nonsingleton nodes, *c*	Density, *D*	Size of the maximal clique, *z*	No. of cliques, *n*
0	2,705	0.998	2,704	10
1	2,705	0.989	2,701	Not available
2	2,705	0.513	860	Not available
3	2,680	0.079	339	1,662,785
4	2,378	0.019	211	6,181
5	2,091	0.008	124	3,344
6	1,860	0.005	82	525
7	1,676	0.003	64	229
8	1,538	0.003	61	224
9	1,358	0.002	48	232

**FIG 2:**
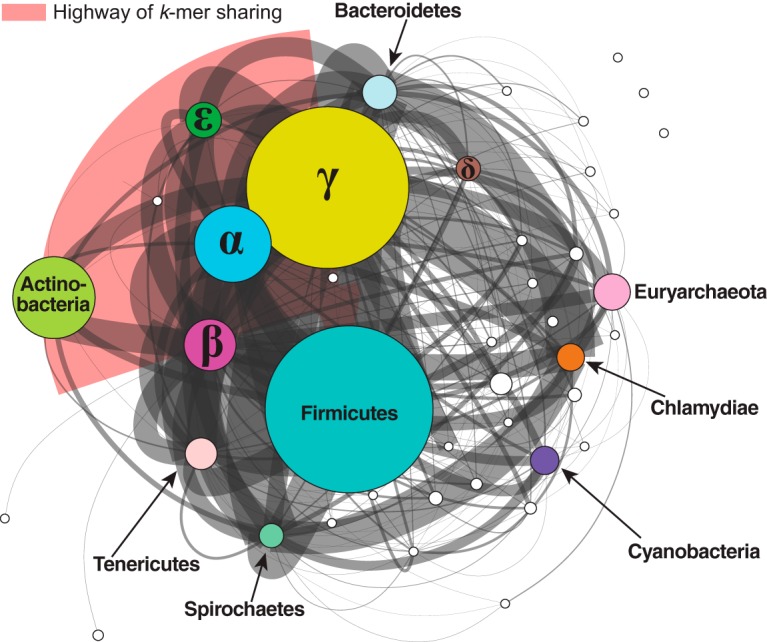
*P*-network of 2,705 prokaryote genomes based on whole-genome data. The network was generated using *D*_2_^*S*^ with *k *=* *25 at *t *=* *3. Each node represents a distinct phylum (or proteobacterial group), with major representative nodes labeled. Each edge between two nodes represents the number of genome pair connections between the two nodes. The thickness of each edge is proportional to the number of genome pairs with shared *k*-mers. The size of each node is proportional to the number of isolates within the phylum. The five representative *Proteobacteria* groups are labeled with the corresponding Greek characters. The highway of *k*-mer sharing between *Betaproteobacteria* and *Gammaproteobacteria* is indicated in red. A dynamic view of this figure is available at http://bioinformatics.org.au/tools/AFmicrobes/.

The *I*-network is very densely connected at *t *=* *0, with the maximum number of cliques *n* = 10. The value *n* is too great to be computed at *t* =1 or *t *=* *2, but *n* = 1,662,785 at *t *=* *3 and decreases to 232 at *t *=* *9 (Table 1). Most isolates are members of a single large clique at *t *=* *0 and *t *=* *1 (*D *>* *0.98 in both cases); at *t *=* *2, *D *=* *0.513. The network becomes less dense at *t *=* *3 (*D *=* *0.079; Table 1). As this network of 2,705 nodes remains too densely connected to be visualized and analyzed directly, we generated the *P*-network using the same data, with each node representing a phylum. Figure 2 shows the *P*-network of the 2,705 genomes at *t *=* *3 (dynamic view available at http://bioinformatics.org.au/tools/AFmicrobes/). The width (thickness) of each edge represents the number of instances in which any two genomes (one from each phylum connected by the edge) have similarity *S*≥*t*; the width is relative to the number of connected genome pairs between two phyla. Major phyla (e.g., *Betaproteobacteria* and *Gammaproteobacteria*, *Firmicutes*, *Actinobacteria*, and *Tenericutes*) are clearly separated at *t *=* *3. The thickest edge (in red) is between the *Betaproteobacteria* and *Gammaproteobacteria* (7,568 connected genome pairs; see Fig. S1A in the supplemental material), suggesting a high similarity among genomes between these groups. In addition, we also observed a large proportion of shared 25-mers between *Firmicutes* and each of the proteobacterial classes.

**FIG 3:**
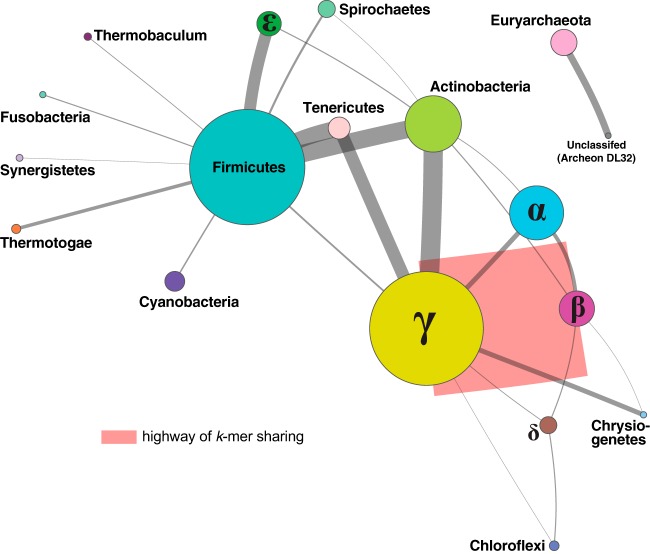
*P*-network of 2,616 prokaryote genomes based on chromosomal sequences with rRNA genes removed. The network was generated using *D*_2_^*S*^ with *k *=* *25 at *t *=* *3; only nonsingleton nodes are shown. Each edge between two nodes represents the number of connections between isolates from the two phyla; the thickness of each edge is proportional to the number of genome pairs with shared *k*-mers. The size of each node is proportional to the number of isolates within the phylum. Singletons are not shown. The five representative *Proteobacteria* groups are labeled with the corresponding Greek characters. The highway of *k*-mer sharing between *Betaproteobacteria* and *Gammaproteobacteria* is indicated in red. A dynamic view of this figure is available at http://bioinformatics.org.au/tools/AFmicrobes/.

10.1128/mSystems.00257-18.2 FIG S1 Number of pair-wise genome connections (relative edge widths) between the phyla in each pair for the networks shown in (A) Fig. 2 (only five most abundant pairs are labeled) and (B) Fig. 3. Download FIG S1, PDF file, 0.4 MB. Copyright © 2018 Bernard et al. 2018 Bernard et al. This content is distributed under the terms of the Creative Commons Attribution 4.0 International license.

### Phylogenomic signal contributed by rRNA genes.

To determine the contribution of the rRNA genes to our AF networks, we first excluded from our set of 2,705 unique genomes (see above) the 89 genomes that did not have gene annotation, and we excluded from the remaining 2,616 all rRNA gene sequences based on annotated start and stop coordinates (see Materials and Methods). The density of the *I*-network of genomes from which rRNA genes have been removed was lower than in the *I*-network inferred using the whole data set. Similarly to what we observed for the *I*-networks described in the previous section, here, at *t *=* *0, *c *=* *2,615 and *z *=* *1,226, and these values decreased to *c *=* *1,290 and *z *=* *47 at *t *=* *9 (Table 2). At *t *=* *3, the *I*-network of the rRNA gene-free network had a network density of *D *=* *0.026, 3-fold lower than the *D *=* *0.079 in the whole-genome network (Table 1). Figure 3 shows the *P*-network of these 2,616 genomes at *t *=* *3 (dynamic view available at http://bioinformatics.org.au/tools/AFmicrobes/). As in Fig. 2, the thickest edge (in red), between *Betaproteobacteria* and *Gammaproteobacteria* (Fig. 3), indicates the largest number of instances of shared *k*-mers between genomes from these two groups. This *P*-network is less dense than the equivalent network based on the whole data set (shown in Fig. 2). Although we observed fewer connections between phyla after removal of rRNA sequences from the genome data, many of the major connections observed in Fig. 2 remained, e.g., the connections between *Betaproteobacteria* and *Gammaproteobacteria* (404 connected genome pairs) and between *Actinobacteria* and *Gammaproteobacteria* (57 connected genome pairs) (see Fig. S1B). Thus, the sharing of 25-mers contributing to these major connections extends beyond the rRNA genes commonly used as phylogenetic markers.

**TABLE 2:** Characteristics of the phylogenomic network of 2,616 prokaryote genomes based on complete genomes without rRNA genes

Threshold	No. of nonsingleton nodes, *c*	Density, *D*	Size of the maximal clique, *z*	No. of cliques, *n*
0	2,615	0.490	1,226	Not available
1	2,597	0.219	548	Not available
2	2,555	0.072	367	164,221
3	2,394	0.026	220	5,379
4	2,182	0.012	159	5,139
5	1,959	0.006	117	631
6	1,761	0.004	74	299
7	1,591	0.003	62	120
8	1,460	0.003	59	117
9	1,290	0.002	47	131

A network computed using only the rRNA sequences was denser than the two corresponding I-networks described above. At *t *=* *6, *D* was high at 0.635 (*z *=* *1,321; see Table S1 in the supplemental material) compared to 0.005 (*z *=* *82) and 0.004 (*z *=* *74) in the *I*-networks based on whole-genome and rRNA gene-removed data, respectively. Figure S2 shows the *P*-network of 2,616 genome isolates based solely on rRNA genes at *t *=* *6. Although almost all phyla were connected to each other (*c *=* *2,613 and *z *=* *1,321 at *t *=* *6), we observed a clear separation between the *Archaea* and *Bacteria*. These results imply that rRNA gene sequences contain sufficient information to distinguish *Archaea* from *Bacteria* by the use of a *k*-mer approach, but separation of bacterial phyla would require further tuning of *k* and *t*.

10.1128/mSystems.00257-18.3 FIG S2 *P*-network of 2,616 prokaryote genomes using *D*_2_^*S*^ with *k *=* *25 based on rRNA genes only, at *t *=* *6. Each edge between two nodes represents one or more connections between isolates from the two phyla. Archaeal phyla (labeled) are clearly separated from bacterial phyla. Download FIG S2, PDF file, 2.1 MB. Copyright © 2018 Bernard et al. 2018 Bernard et al. This content is distributed under the terms of the Creative Commons Attribution 4.0 International license.

10.1128/mSystems.00257-18.6 TABLE S1 Characteristics of the phylogenomic network of 2,616 prokaryote genomes based on rRNA genes only. Download Table S1, PDF file, 0.03 MB. Copyright © 2018 Bernard et al. 2018 Bernard et al. This content is distributed under the terms of the Creative Commons Attribution 4.0 International license.

### Phylogenomic signal contributed by plasmid genomes.

**FIG 4:**
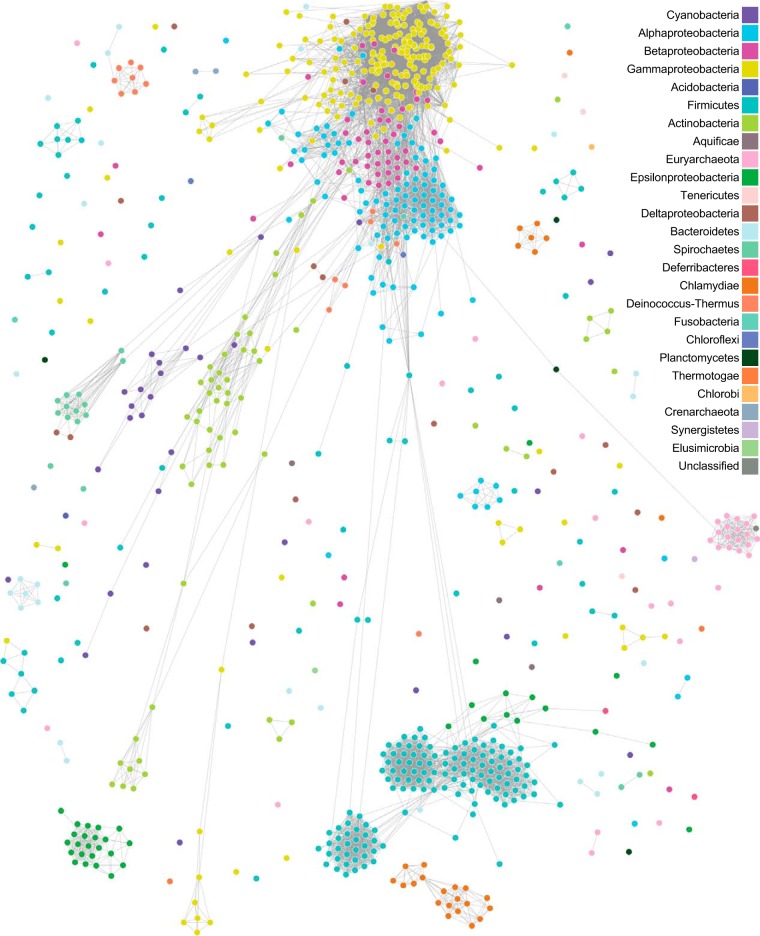
*I*-network of 921 plasmid genomes. The network was generated using *D*_2_^*S*^ with *k *=* *25 at *t *=* *0. Each edge between two nodes represents evidence of shared *k*-mers. A dynamic view of this figure is available at http://bioinformatics.org.au/tools/AFmicrobes/.

Among the genome data records available to this study, 921 (representing 26 phyla) include sequence annotated as arising from one or more extrachromosomal plasmids. To examine the phylogenomic signal contributed by these plasmids, we computed *I*- and *P*-networks using only the plasmid sequences for these 921 isolates (see Materials and Methods). Figure 4 shows the *I*-network of the 921 plasmid genomes at *t *=* *0, in which *D = *0.025 (*c *=* *745 and *z *=* *48; Table 3); a dynamic view is available at http://bioinformatics.org.au/tools/AFmicrobes/. Most phyla appear as distinct cliques, but, notably, there are edges between *Proteobacteria* and *Actinobacteria* and between *Proteobacteria* and *Firmicutes*. At *t *=* *4, most phyla are separated as distinct cliques, with the exception of *Epsilonproteobacteria* and *Firmicutes*; the other *Proteobacteria* (*Alphaproteobacteria*, *Betaproteobacteria*, *Deltaproteobacteria*, and *Gammaproteobacteria*) are in a distinct paraclique. The *Euryarchaeota*, connected only to the bacterial phylum *Planctomycetes* at *t *=* *0, is separated from *Bacteria* at *t *≥* *1. All phyla are disjoint at *t *=* *7. These results are not surprising, as the plasmid genomes can have a narrow host range (38, 39) and are known to evolve faster than the core genomes (40); in combination with their smaller genome size, fewer shared *k*-mers are observed at a given similarity threshold (41).

**TABLE 3:** Characteristics of the phylogenomic network of 921 prokaryote genomes based on plasmid sequences only

Threshold	No. of nonsingleton nodes, *c*	Density, *D*	Size of the maximal clique, *z*	No. of cliques, *n*
0	745	0.025	48	20,557
1	718	0.021	46	13,272
2	680	0.017	45	3,925
3	648	0.014	39	1,406
4	601	0.011	34	800
5	556	0.009	30	589
6	499	0.006	25	368
7	439	0.004	13	122
8	353	0.002	11	26
9	245	0.001	9	14

For each genome pair, we further compared its *D*_2_^*S*^ distance derived from whole genome data set to those derived from distinct genome components (Fig. S3). Distances derived from rRNA sequences are almost always smaller than the distances derived from the overall data set. The reverse trend is observed for distances derived from chromosomal sequences with rRNAs removed (although a one-to-one relationship is observed) and to a greater extent for those derived from plasmid sequences.

10.1128/mSystems.00257-18.4 FIG S3 Relationship of pairwise *D*_2_^*S*^ distances derived from whole-genome data sets with those derived from distinct genome components. The composite plot is shown (A); individual plots are shown for (B) chromosomal sequences with rRNAs removed, (C) rRNA sequences, and (D) plasmid sequences. Download FIG S3, PDF file, 2.4 MB. Copyright © 2018 Bernard et al. 2018 Bernard et al. This content is distributed under the terms of the Creative Commons Attribution 4.0 International license.

### Network comparison.

**FIG 5:**
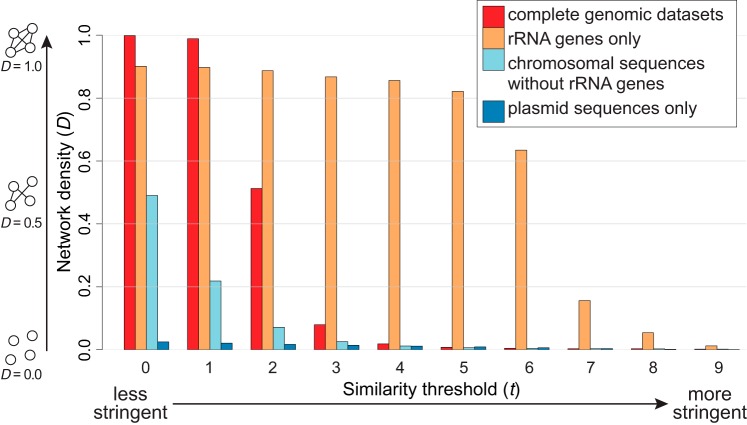
Density of alignment-free phylogenomic networks. Network density (*D*) across distinct threshold levels of *t* is shown for each *I*-network based on complete genomic data sets (core genomes with rRNAs plus plasmids), rRNA genes only, chromosomal sequences without rRNA genes, and plasmid sequences only. The density of a four-node network is illustrated for *D *=* *0.0, 0.5, and 1.0 on the left, and the stringency of the threshold *t* is shown at the bottom.

Figure 5 shows the density *D* for all four *I*-networks as a function of threshold *t*. For all networks, the network density decreases as *t* increases. At *t *>* *2, the rRNA gene-only network is denser than the others, with *D* remaining >0.63 through *t *=* *6, compared to *D *<* *0.02 for the others at *t > *3. As expected, the highest density of the complete-genome network is observed at *t *<* *2; *D *>* *0.98 and decreases rapidly at 2 < *t *<* *5. The network without rRNA genes exhibits a lower density, *D *<* *0.5, at *t *=* *0, and by *t *=* *5, *D* has decreased to a level similar to that calculated for the complete-genome network (*D *<* *0.01). Together with our observed pairwise genome distances based on distinct genome components (Fig. S3), these results confirm that rRNA sequences (as captured by 25-mers) are more highly conserved than are the genome sequences overall. The data corresponding to the whole-genome and rRNA-free networks differ through a similar range of network densities, whereas data corresponding to the rRNA gene network differ at a higher threshold (i.e., *t *>* *5). The plasmid network shows the lowest density, with *D *<* *0.03 at *t *≥* *0 (Fig. 5), indicating that these plasmid genomes are more diverse in 25-mer composition than are the corresponding main genomes. The results presented in Fig. 5 provide a guide for visualization and comparison of these networks at the appropriate *t* values. In this study, we chose *t* values that would yield a clear separation of *Bacteria* and *Archaea*; thus, we used *t *=* *3 for visualizing the two networks shown in Fig. 2 and 3 (i.e., the use of the same *t* value for both networks is purely coincidental) and *t *=* *0 for the plasmid network shown in Fig. 4.

**FIG 6:**
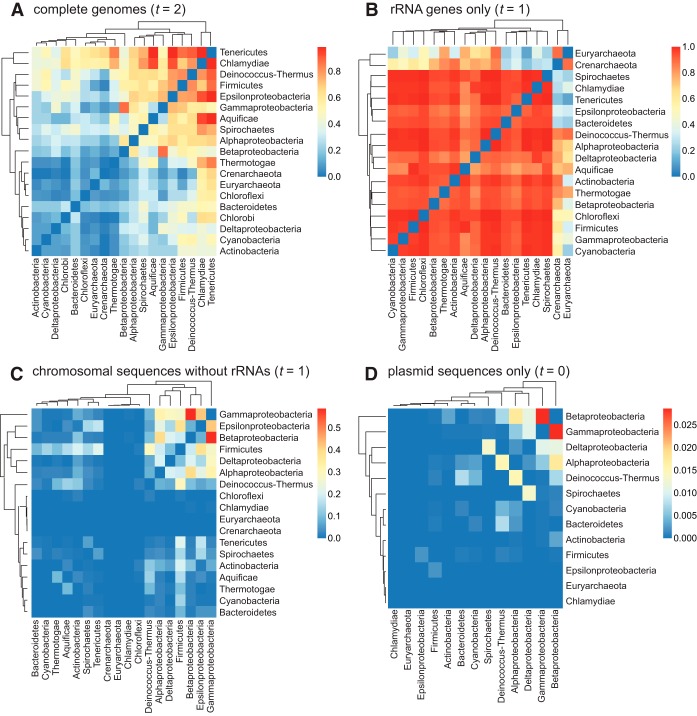
Phylum connectedness based on shared *k*-mers. Summary data representing phylum connectedness (*C*) in a heat map for each *P*-network reconstructed based on (A) complete genomic data sets at *t *=* *2, (B) rRNA gene sequences only at *t *=* *1, (C) chromosomal sequences without rRNA genes at *t *=* *1, and (D) plasmid sequences only at *t *=* *0 are shown.

To assess the (individual) contributions of rRNA genes and plasmids to the relatedness among the distinct phyla, we calculated for each phylum pair a connectedness value *C*, representing the proportion of genome pairs that share one or more *k*-mers over all possible genome pairs in the two phyla (see Materials and Methods). As shown in the heat map summaries (Fig. 6), the hierarchical clustering of *C* values does not conform to known phyletic relationships; e.g., proteobacterial groups are not unified a single cluster. In the all-inclusive genome network (Fig. 6A), the archaeal phyla (*Crenarchaeota* and *Euryarchaeota*) are not clearly separated from the *Bacteria* phylum and show substantial connectedness with *Tenericutes* (*C *>* *0.63) and *Chlamydiae* (*C *>* *0.54). The highest mean *C* value (0.85) was observed in the network consisting only of rRNA genes (Fig. 6B), with *Archaea* and *Bacteria* clearly separated. *Crenarchaeota* shows substantial connectedness (*C *>* *0.5) with 11 bacterial phyla, compared to *Euryarchaeota* with 6; both cases include *Deinococcus*-*Thermus*, *Aquificae*, and *Thermotogae*. The removal of rRNA genes from the genome sequences appears to have removed most of the connectedness among phyla (mean *C *=* *0.05 in Fig. 6C), with the maximum *C *=* *0.59 between *Betaproteobacteria* and *Gammaproteobacteria*. Even less phylum-level connectedness was observed in the plasmid-only network (Fig. 6D; mean *C *=* *0.002), with maximum *C *=* *0.029 between *Betaproteobacteria* and *Gammaproteobacteria*. These results indicate the complications of inferring a tree-like structure among these taxa using genome-wide *k*-mers and that whole-genome and plasmid sequences capture phyletic relatedness that is distinct from that captured by the rRNA genes. Remarkably, chromosomal sequences, apart from rRNA genes, although usually representing more than 99% of the genome sequences, contribute little to overall phylogenetic signal.

### Core *k*-mers of microbial genera.

We define a core *k*-mer in a group of interest as a *k*-mer that is present in every genome within the group, e.g., a core 25-mer in *Proteobacteria* is present in all proteobacterial genomes in our database (see Materials and Methods). We identified core 25-mers for each genus in our 2,783-genome data set. Of these 699 genera, 497 are represented by only a single genome isolate, and a further 51 consist of highly divergent genomes for which no core 25-mers were identified; we exclude these data from this part of analysis. The remaining 151 genera for which core 25-mers were identified are listed in Table S2. As these genera are represented in our data set by different numbers of isolates, we define *K* as the number of distinct core *k*-mers per isolate for each genus; this value can help describe the extent of genome divergence (and thus the evolutionary rate of these genomes) within each of these genera. Thus, the three genomes representing genus *Azotobacter* show the highest number of core *k*-mers, and *K *=* *1,722,079; these genomes represent distinct isolates of the same species, Azotobacter vinelandii. This is in contrast to the 123 *Streptococcus* genomes (in 27 described species), which share only one core *k*-mer (*K *=* *0.01). Among the 20 genera with the greatest *K* values, *Shigella* is represented here by the greatest number of distinct isolates (10 from four species), and *K *=* *33,698. This number compares to *K *=* *4.82 among the 11 *Ralstonia* genome isolates from three species. Thus, these *Shigella* genomes have diverged much less from their common ancestor than have these *Ralstonia* genomes from theirs, as assessed by shared 25-mers. This result also lends support to the earlier discovery of extensive gene dispersal among six genomes of Ralstonia solanacearum (of the 11 *Ralstonia* isolates in our data set) (42).

10.1128/mSystems.00257-18.7 TABLE S2 Core *k*-mers identified in 151 genera of prokaryotes. Download Table S2, PDF file, 0.1 MB. Copyright © 2018 Bernard et al. 2018 Bernard et al. This content is distributed under the terms of the Creative Commons Attribution 4.0 International license.

### Core functions of microbial phyla.

To relate the shared *k*-mers to biological functions, for all 25-mers in these 2,783 genomes we organized the genome coordinates of each instance, and the biological function annotated for the gene product encoded at those coordinates, in a relational database. Functional annotation was based on Clusters of Orthologous Groups (COGs) (43). Then, using the list of core 25-mers described above, we grouped these 25-mers by taxon, focusing on protein-coding sequences (i.e., rRNA sequences were discarded; see Materials and Methods). This yielded a set of core 25-mers for 112 genera in 16 phyla; the corresponding COG functional categories for these core 25-mers are shown in Table S3. The noninformative functional categories R (general function prediction only) and S (function unknown) were excluded from subsequent analyses. No core *k*-mer in our data set was found to be associated with functional category Y (nuclear structure). Functional categories represented at <1% of core *k*-mers in each genus included category A (RNA processing and modification), category B (chromatin structure and dynamics), category W (extracellular structure), and category Z (cytoskeleton).

10.1128/mSystems.00257-18.8 TABLE S3 Number of core *k*-mers in 112 genera of prokaryotes, based on their annotated function in COG functional categories. Download Table S3, PDF file, 0.2 MB. Copyright © 2018 Bernard et al. 2018 Bernard et al. This content is distributed under the terms of the Creative Commons Attribution 4.0 International license.

**FIG 7:**
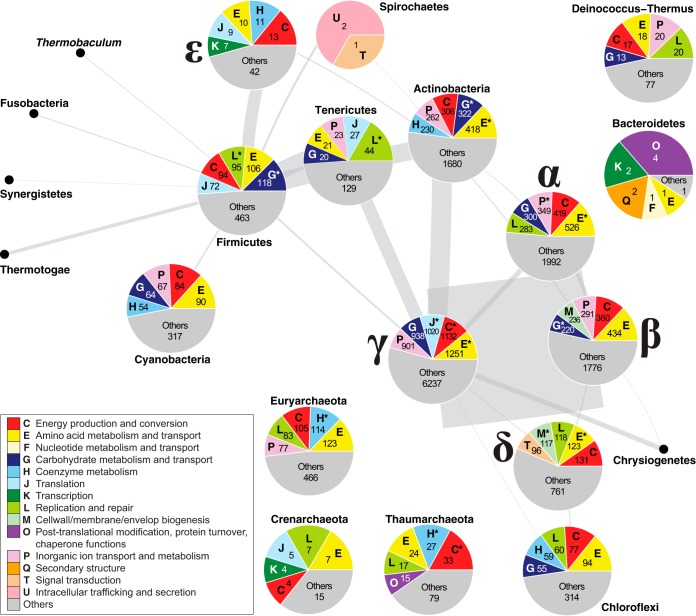
Functions of core *k*-mers in microbial taxa. A *P*-network of 2,616 prokaryote genomes using *D*_2_^*S*^ with *k *=* *25 based on chromosomal sequences with rRNA genes removed at *t *=* *3 is shown. At each node (phylum) where core *k*-mers are available, a pie chart representing the COG categories annotated for these core *k*-mers is shown. Only the top five COG categories and the corresponding numbers of core *k*-mers are shown for each phylum; in most cases, the top five categories account for >50% of the annotated core *k*-mers. The categories that are significantly enriched in a phylum (*P ≤ *0.05) are noted with an asterisk (*). Each edge between two nodes represents the number of connections between isolates from the two phyla; the thickness of each edge is proportional to the number of shared *k*-mers. The five representative *Proteobacteria* groups are labeled with the corresponding Greek characters.

We found core *k*-mers associated with functional category A only in the proteobacterial classes *Alphaproteobacteria*, *Betaproteobacteria*, *Gammaproteobacteria*, and *Deltaproteobacteria* (i.e., not in the *Epsilonproteobacteria*) and in phylum *Actinobacteria* and those associated with functional category B only in phyla *Chloroflexi*, *Euryarchaeota* and *Thaumarchaeota*. Figure 7 shows the proportions of the five most-frequent COG categories associated with core 25-mers across the 23 COG categories for 16 phyla. Categories E (amino acid metabolism and transport) and C (energy production and conversion) are among the five most abundant categories in 15 and 13 phyla, respectively. The *Epsilonproteobacteria*, *Thaumarchaeota*, *Euryarchaeota*, *Actinobacteria*, *Cyanobacteria*, and *Chloroflexi* represent the only phyla with category H (coenzyme metabolism) among the five most abundant. For the phyla *Tenericutes*, *Deinococcus*-*Thermus*, *Firmicutes* and *Crenarchaeota*, the most-represented functional categories include P (inorganic ion transport and metabolism), L (replication and repair), J (translation), E (amino acid transport and metabolism), and G (carbohydrate metabolism and transport). *Bacteroidetes* is the only phylum for which categories O (posttranslational modification, protein turnover, and chaperone functions), Q (secondary structure), and F (nucleotide metabolism and transport) are among the top five. Phylum *Spirochaetes* is the only one with U (intracellular trafficking and secretion) and T (signal transduction) among the five most abundant, but very few COGs are associated with core 25-mers.

Comparing the annotated core *k*-mers in each phylum to all annotated core *k*-mers, 11 of the 25 COG functional categories are significantly enriched in *Gammaproteobacteria* (Fisher’s exact test, Benjamini-Hochberg [44]-adjusted *P ≤ *0.05), 9 in *Alphaproteobacteria*, 9 in *Actinobacteria*, 8 in *Deltaproteobacteria*, and 7 in *Firmicutes* (Table S4). This observation may be due to the large (73.6%) representation of taxa of these phyla in the overall 2,783 data set: 1,163 (41.8%) *Proteobacteria*, 601 (21.6%) *Firmicutes*, and 285 (10.2%) *Actinobacteria* (Data Set S1). In comparison, category L (replication and repair) is enriched (*P = *7.55 × 10^−6^) among the core *k*-mers of *Tenericutes* and category M (cell wall/membrane/envelop biogenesis; *P = *4.88 × 10^−9^) in *Euryarchaeota*. These results suggest a more prominent conservation of these functions in these phyla than in the others, indicating their importance.

10.1128/mSystems.00257-18.9 TABLE S4 Comparison of annotated core *k*-mers in each phylum against all annotated core *k*-mers, based on COG functional categories. Download Table S4, PDF file, 0.1 MB. Copyright © 2018 Bernard et al. 2018 Bernard et al. This content is distributed under the terms of the Creative Commons Attribution 4.0 International license.

In order to determine whether the phyla can be clustered based on their COG-category profiles, we performed a series of principal-component analyses (PCA). PCA of the raw data (e.g., of nonnormalized counts of COG number) did not reveal any particular clustering (Fig. S4A), nor did PCA of the clusters of genera classified according to the number of isolates (Fig. S4B). Figure S4C shows the results of PCA performed on the normalized counts of COG numbers in a centered scale (e.g., COG categories with equal weights). In this analysis, *Nitrosopumilus*, the only genus in phylum *Thaumarchaeota* represented in this data set, is isolated from the other genera, as is genus *Dehalococcoides*, a member of phylum *Chloroflexi*. These results confirm that the different numbers of isolates per genus do not bias our analysis of functional categories but that some phyla can be distinguished from others.

10.1128/mSystems.00257-18.5 FIG S4 Principal-component analysis (PCA) of core *k*-mers and their annotated COG categories, based on core *k*-mers in each (A) phylum and (B) genus. Results of PCA of core *k*-mers in each phylum performed on the normalized counts of COG categories in the centered scale are shown (C). Download FIG S4, PDF file, 0.7 MB. Copyright © 2018 Bernard et al. 2018 Bernard et al. This content is distributed under the terms of the Creative Commons Attribution 4.0 International license.

## DISCUSSION

Phylogenetic studies have long been based on multiple-sequence alignment (thus the implicit assumption of full-length contiguity), from which a phylogenetic tree is inferred. A tree-like structure is an unrealistic representation of microbial evolution due to complications of horizontal signal caused by genome rearrangements and lateral genetic transfer (33, 45, 46). In this study, we demonstrated that AF approaches can be used to infer phylogenetic networks quickly for large-scale microbial whole-genome data (see also Text S1 in the supplemental material). Our results provide a comprehensive, alignment-free view of microbial genome evolution as a network, beyond a tree-like structure. We introduce for the first time the concept of a *k*-mer similarity network and two types of AF networks, the *I*- and *P*-networks. We show that by combining a *k*-mer approach with the use of a relational database, biological information can be accessed efficiently for large-scale data. Finally, we define core *k*-mers as consisting of those *k*-mers present in every isolate genome of a genus (or other taxon), following the concept of core genes (47, 48).

10.1128/mSystems.00257-18.1 TEXT S1 Computational scalability and runtime of AF phylogenomics. Download Text S1, PDF file, 0.2 MB. Copyright © 2018 Bernard et al. 2018 Bernard et al. This content is distributed under the terms of the Creative Commons Attribution 4.0 International license.

We examined the contributions of rRNA genes and plasmids to the phylogenomic signal among microbial genomes. As expected, rRNA genes contribute to the signal captured by 25-mers, as they do in MSA-based approaches. However, the pattern of network density versus threshold (Fig. 5) clearly indicates the different extents of sequence conservation in the distinct genomic regions. Our demonstration that, in general, the signal contributed by rRNA genes is not by itself sufficient to resolve relationships among (and sometimes within) bacterial phyla is in line with many previous studies (2, 6, 49, 50). The low density of the plasmid *k*-mer network also confirms that plasmids tend to be taxon specific (41). In all our AF networks, phyletic relatedness based on shared *k*-mers is often strongest between proteobacterial classes, in particular, between the *Betaproteobacteria* and *Gammaproteobacteria*, and many 25-mers are shared between the *Actinobacteria* and *Proteobacteria* or *Firmicutes* across all networks. Lateral genetic transfer between lineages of *Betaproteobacteria* and *Gammaproteobacteria* (14), identified in earlier studies based on MSA (14, 51) and *k*-mers (11), partly explains this strong similarity in our networks.

Overall, the *I*- and *P*-networks provide a quick overview of the evolutionary relationships among whole genomes, or subsets of genomes, in large-scale data sets. The *I*-networks capture evolutionary dynamics (e.g., divergence and lateral genetic transfer) and relatedness among individual genomes, providing a fine-scale overview of shared genetic elements among these genomes. The *P*-networks capture phyletic relatedness and illustrate the magnitude of the sharing of *k*-mers (and genetic elements) among these groups at a deeper evolutionary timescale.

Assignment of taxonomic rank to groups of bacteria has long been considered fraught (52–54), and there is no generally accepted way to extract taxonomic rank from trees. This undertaking is further complicated by the imbalance in the number of isolates per higher taxon. Our *k*-mer similarity networks provide an alternative way to explore the evolutionary dynamics of microbial genomes that tracks taxonomic rank. In our phylogenomic network based on 2,705 complete genomic data sets, at threshold at *t *<* *3, domains *Archaea* and *Bacteria* appear as separate regions of dense connection within the AF graph. At 3 ≤ *t* ≤ 5, phyla (e.g., *Proteobacteria* and *Firmicutes*) emerge. We see classes (e.g., of *Proteobacteria*) at 4 ≤ *t* ≤ 6 and structure between and/or within genera (e.g., Escherichia coli and *Shigella*) at *t *>* *6. Our *k*-mer phylogenomic network allows dynamic genome-scale exploration of the taxonomic rank.

Relating the identified core 25-mers for each genus to annotated functions of the corresponding genes identifies highly conserved functions. Although we took great care to use only 2,873 completely sequenced and annotated prokaryote genomes (and excluded draft, fragmented genomes with suboptimal annotations), we cannot dismiss entirely the possible impact of technical errors or inconsistencies of the genome annotation process (e.g., due to chains of functional inference) on these data sets. However, the annotated functions of core *k*-mers represent conservation at a finer scale than those based on full-length sequence comparisons and remain biologically relevant. Across the phyla represented in our data set, functions (identified on this basis) associated with the metabolism and transport of amino acids, and with the production and conversion of energy, are the ones most frequently encountered. Perhaps not surprisingly, we observed that phyla that share many 25-mers also exhibit similar core functional profiles. Our analysis reveals that the core functions highly conserved in *Epsilonproteobacteria* and in *Deltaproteobacteria* are distinct from those conserved in the other proteobacterial classes. Except for the two most highly conserved categories (see above), the *Epsilonproteobacteria* do not share highly conserved functions with the other classes of *Proteobacteria*; indeed, the *Epsilonproteobacteria* share more 25-mers with the *Firmicutes* and with the *Actinobacteria* than with other *Proteobacteria*. These results support those of previous single-gene phylogenetic analyses revealing *Epsilonproteobacteria* to be the most basal proteobacterial lineage and are consistent with *Epsilonproteobacteria* having been the last class in this phylum to have been recognized (55). Finally, we also observed that phylum *Tenericutes* is among the only phyla that do not have highly conserved functions related to energy production and conversion; this can be related to their parasitic or commensal lifestyle (56). These results demonstrate that analysis of conserved *k*-mers can identify molecular mechanisms and functions that characterize evolutionary diversification within and among microbial taxa.

No core 25-mers were recovered for 51 of these 699 genera, particularly those represented by genome sequences for many isolates from different species. For such genera, a core *k*-mer set might be sought at lower values of *k*, although at the potential risk of including signal from false positives and background noise (i.e., nonhomologous *k*-mers). Similarly, some phyla that we pointed out as sharing highly conserved functions have few distinct COGs related to core 25-mers.

## MATERIALS AND METHODS

### Data.

In total, 2,785 completely sequenced genomes of *Bacteria* and *Archaea* were downloaded from NCBI on 31 January 2016 (Data Set S1); two of these were identified as “multispecies” and “multi-isolate” and were thus excluded. Functional annotation of the remaining 2,783 genomes was obtained through the corresponding RefSeq records. Genes encoding ribosomal RNAs were identified based on annotation. Genomes with no annotation information were excluded from our rRNA-gene network. Of the 2,783 isolates, 921 contained plasmids; these plasmid genomes were used in the plasmid-only network.

### Relational database of *k*-mers and genome features.

We extracted 10,059,526,408 distinct 25-mers from the genomes of 4,401 bacterial and archaeal isolates (present as of 31 January 2016 in NCBI RefSeq), of which 2,783 genomes were complete and included in our subsequent analysis (see above). We organized these *k*-mers, and their genomic locations and features (based on RefSeq annotations), in a relational database using SQL, following the method of Greenfield and Roehm (37). Tables in the database contain a list of isolates, lists of genes and their sequences, coherent taxonomic information for each isolate, an indexed list of all 25-mers, an indexed list of gene-by-gene comparisons for each pair of genes, and an indexed list of genome-by-genome comparisons for each pair of genomes.

### Alignment-free (AF) network.

We followed the method of Bernard et al. (29) in generating the AF networks. We first computed pairwise comparisons for the 2,783 isolates and generated for each comparison the corresponding *D*_2_^*S*^ distance (15) value *d*, using 25-mers across parallel central processing units (CPUs). For a pair of genomes *a* and *b*, we transformed *d* into a similarity measure *S_ab_*, where *S_ab_*=10- *d*. For instance, considering two highly similar genomes of *a* and *b* for which distance *d_ab_* = 0.001, the similarity measure *S_ab_* = 9.999. Likewise, considering two highly dissimilar genomes of *a* and *b* for which *d_ab_* = 9.925, *S_ab_* = 0.075. We ignored any edge for which *d *>* *10 (i.e., for which the *S* value was negative), as the corresponding pair of sequences shares only ≤0.01% of 25-mers (i.e., 25-mers capture little evidence of homology). We then generated the networks using JSON files containing the *S* values as input for a Javascript script using the D3 library (https://d3js.org/). Here, we present two types of AF networks. For a phylum-level depiction of the network (*P*-network), we grouped all sequences of the same phylum as a single entity prior to calculating the distance; each phylum is represented by a node in the network. The width of the edge between two nodes represents the number of connections between isolates from these two phyla, and the size of each node is proportional to the number of isolates in the phylum. For an isolate-level depiction of the network (*I*-network) we treated each genome isolate as a single entity (i.e., node). In this network, an edge between two nodes indicates evidence of shared *k*-mers. The AF networks include a similarity-score threshold *t*, for which only edges with *S*>*t* are displayed; changing *t* therefore can dynamically change the structure of the network (29). The resulting dynamic networks can be visualized using any web browser. All of the networks are available at https://doi.org/10.14264/uql.2017.436.

### Network density and phylum connectedness.

For a network with *x* nodes, there are *e* possible edges (potential connections), where $e=\frac{x(x-1)}{2}$. For a network containing *y* edges (actual connections), the density value *D* was calculated as $\frac{y}{e}$ (Fig. 1). For a pair of phyla *a* and *b*, their connectedness value *C_ab_* is$\;\frac{g}{G}$, where *g* is the number of genome pairs (between phyla *a* and *b*) that share one or more *k*-mers and *G* is the number of all possible genome pairs between phyla *a* and *b*. In this case, *G* = *N_a_* × *N_b_*, where *N_a_* and *N_b_* represent the number of genomes or isolates in phylum *a* and phylum *b*, respectively. For each network, *C* values were calculated at the optimal threshold *t* for which the connectedness signal is neither too strong nor too weak across all phylum pair comparisons. To avoid potential biases of incomplete taxon sampling, here we restricted our comparisons to phyla that have ≥10 genomes.

### Core *k*-mers and COG categories.

For a specific group of microbial isolates (representing, e.g., a genus or a phylum), we extracted the set of the 25-mers that are found in all isolates within the group; we define this set of 25-mers as the core *k*-mers for the corresponding group. Using the relational database of *k*-mers (see above), we identified for these core 25-mers their corresponding genome locations and function based on COG (Clusters of Orthologous Groups) (57) annotations in RefSeq records. We generated profiles of COG functional categories for each of the 151 genera, for each of the 11 phyla, and for the five proteobacterial classes in which core *k*-mers were identified using our approach.
