# Supplementary material for: k-mer Similarity, Networks of Microbial Genomes, and Taxonomic Rank
Source: mSystems. 2018 Nov 20;3(6):e00257-18. doi: 10.1128/mSystems.00257-18 (PMC6247013; doi:10.1128/mSystems.00257-18)
Supplement: FIG S1 [file sys006182296sf1.pdf]

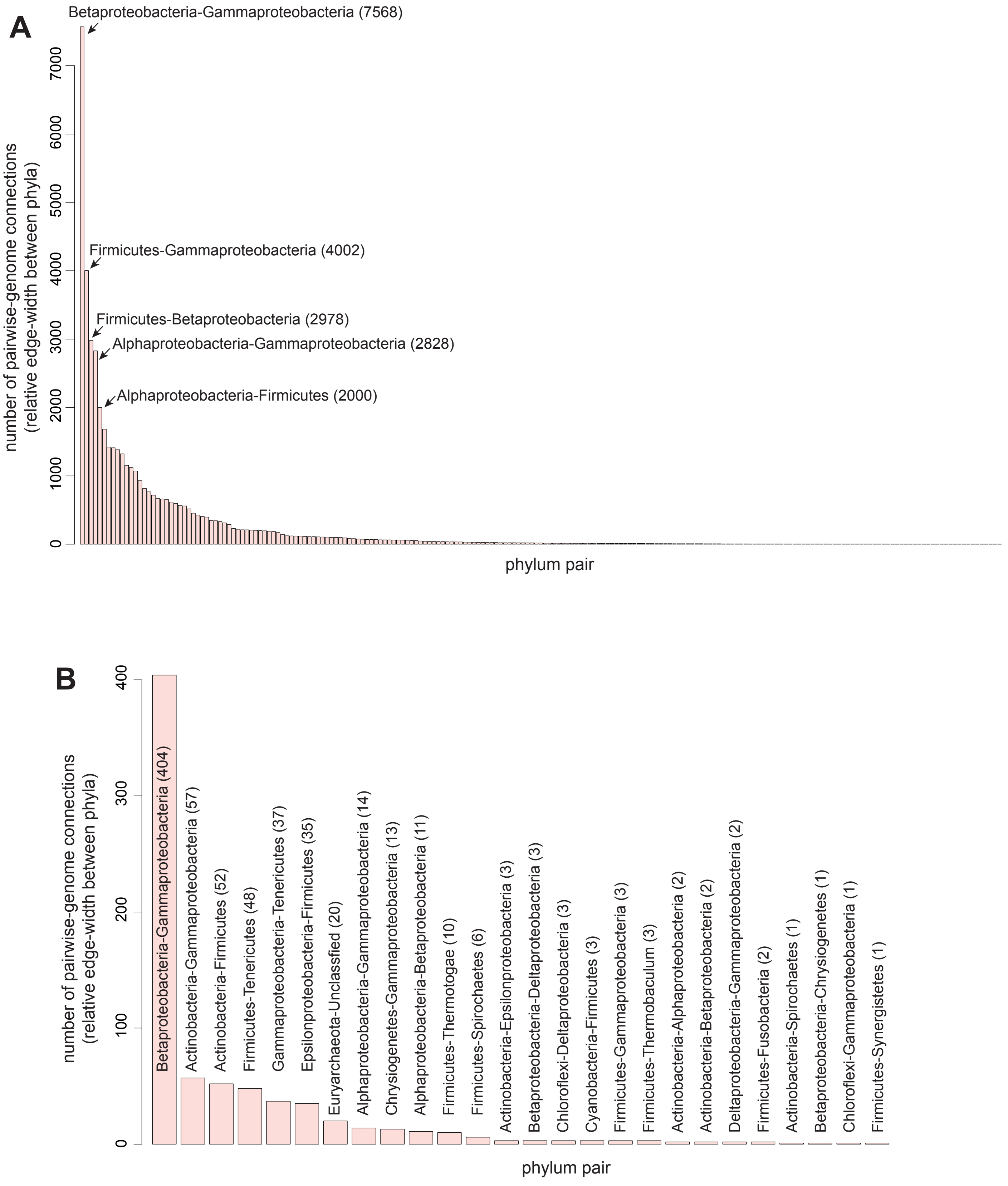

Figure S1. Number of pair-wise genome connections (relative edge width) between each pair of phyla, for the networks shown in (A) Figure 2 (only five most abundant pairs are labelled), and (B) Figure 3.
