# Supplementary material for: k-mer Similarity, Networks of Microbial Genomes, and Taxonomic Rank
Source: mSystems. 2018 Nov 20;3(6):e00257-18. doi: 10.1128/mSystems.00257-18 (PMC6247013; doi:10.1128/mSystems.00257-18)
Supplement: FIG S2 [file sys006182296sf2.pdf]

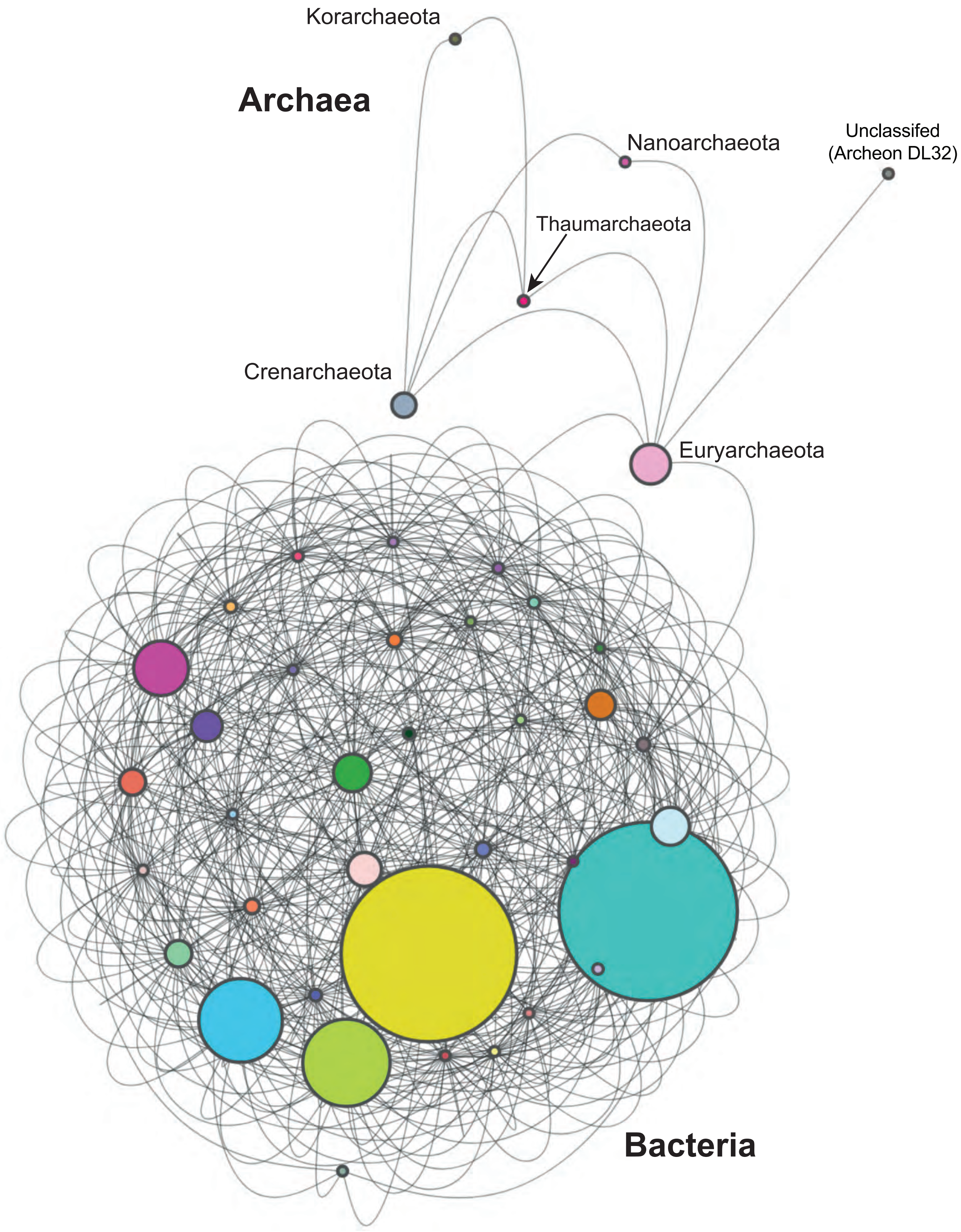

Figure S2. *P*-network of 2616 prokaryote genomes using D_2^S with $k = 25$ based on rRNA genes only, at $t = 6$. An edge between two nodes represents one or more connections between isolates from the two phyla. Archaeal phyla (labelled) are clearly separated from bacterial phyla.
