# Supplementary material for: k-mer Similarity, Networks of Microbial Genomes, and Taxonomic Rank
Source: mSystems. 2018 Nov 20;3(6):e00257-18. doi: 10.1128/mSystems.00257-18 (PMC6247013; doi:10.1128/mSystems.00257-18)
Supplement: TABLE S1 [file sys006182296st1.pdf]

Table S1. Characteristics of the phylogenomic network of 2616 prokaryote genomes based on rRNA genes only.

Threshold	Number of non-singleton nodes, *c*	Density, *D*	Size of the maximal clique, *z*	Number of cliques, *n*
0	2616	0.901	2356	N/A
1	2616	0.897	2356	N/A
2	2616	0.887	2344	N/A
3	2616	0.867	2332	N/A
4	2616	0.856	2291	N/A
5	2616	0.821	2045	N/A
6	2613	0.635	1321	N/A
7	2597	0.156	530	N/A
8	2509	0.054	299	45272
9	2162	0.012	185	289
