# Supplementary material for: k-mer Similarity, Networks of Microbial Genomes, and Taxonomic Rank
Source: mSystems. 2018 Nov 20;3(6):e00257-18. doi: 10.1128/mSystems.00257-18 (PMC6247013; doi:10.1128/mSystems.00257-18)
Supplement: FIG S3 [file sys006182296sf3.pdf]

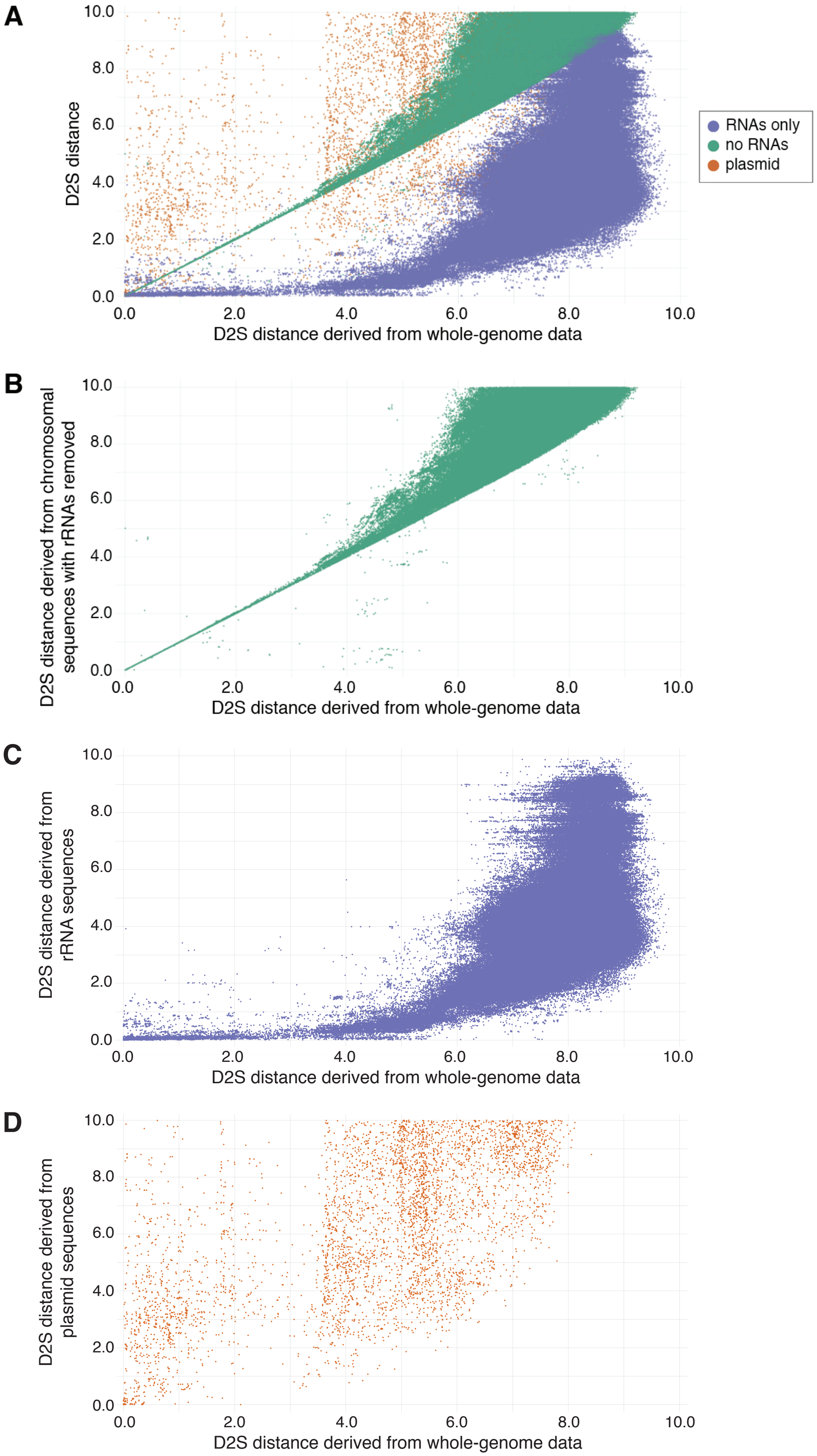

Figure S3. Relationship of pairwise D_2^S distances derived from whole-genome datasets with those derived from distinct genome components. The composite plot is shown in (A), with the individual plot is shown for (B) chromosomal sequences with rRNAs removed, (C) rRNAs sequences, and (D) plasmid sequences.
