# Supplementary material for: k-mer Similarity, Networks of Microbial Genomes, and Taxonomic Rank
Source: mSystems. 2018 Nov 20;3(6):e00257-18. doi: 10.1128/mSystems.00257-18 (PMC6247013; doi:10.1128/mSystems.00257-18)
Supplement: TABLE S2 [file sys006182296st2.pdf]

Table S2. Core *k*-mers identified in 151 genera of prokaryotes.

Genus	Number of distinct core *k*-mers	Number of isolates	Number of core *k*-mers per isolate, *K*
Azotobacter	5166237	3	1722079.00
Synechocystis	3526700	6	587783.33
Rahnella	1053800	3	351266.67
Acetobacter	2042974	9	226997.11
Haloarcula	564756	3	188252.00
Edwardsiella	643366	4	160841.50
Hydrogenobaculum	482260	3	160753.33
Xylella	627213	5	125442.60
Stenotrophomonas	384359	4	96089.75
Clavibacter	245101	3	81700.33
Variovorax	232615	3	77538.33
Pasteurella	201523	4	50380.75
Ureaplasma	141564	3	47188.00
Shigella	336980	10	33698.00
Achromobacter	87624	3	29208.00
Phaeobacter	79509	3	26503.00
Myxococcus	72867	3	24289.00
Amycolatopsis	113989	5	22797.80
Cupriavidus	65493	3	21831.00
Coxiella	104030	5	20806.00
Pectobacterium	76126	5	15225.20
Thermoanaerobacterium	39395	3	13131.67
Nocardia	36570	3	12190.00
Azospirillum	28797	3	9599.00
Anaeromyxobacter	31770	4	7942.50
Meiothermus	21977	3	7325.67
Aeromonas	24147	4	6036.75
Gordonia	17512	3	5837.33
Actinoplanes	20813	4	5203.25
Roseburia	14944	3	4981.33
Aggregatibacter	18551	4	4637.75
Caulobacter	16487	4	4121.75
Dehalococcoides	31804	8	3975.50
Desulfitobacterium	15811	4	3952.75
Chloroflexus	9267	3	3089.00
Pediococcus	8560	3	2853.33
Gluconacetobacter	8177	3	2725.67
Desulfosporosinus	7832	3	2610.67
Nitrosopumilus	7442	3	2480.67
Carnobacterium	7435	3	2478.33
Erwinia	15869	7	2267.00
Oligotropha	6700	3	2233.33
Cronobacter	10482	5	2096.40
Sinorhizobium	20194	10	2019.40
Riemerella	9742	5	1948.40
Acidovorax	9544	5	1908.80
Mesorhizobium	7575	4	1893.75
Methanosarcina	7548	4	1887.00
Marinomonas	5524	3	1841.33
Dickeya	7131	4	1782.75
Psychrobacter	7024	4	1756.00
Anabaena	4613	3	1537.67
Taylorella	7201	5	1440.20
Agrobacterium	5343	4	1335.75

Genus	Number of distinct core *k*-mers	Number of isolates	Number of core *k*-mers per isolate, *K*
Exiguobacterium	5335	4	1333.75
Arcobacter	6665	5	1333.00
Marinobacter	4554	4	1138.50
Nitrosococcus	3395	3	1131.67
Rhodococcus	6153	6	1025.50
Actinobacillus	4920	5	984.00
Pantoea	5889	6	981.50
Pseudoalteromonas	2877	3	959.00
Liberibacter	3637	4	909.25
Nostoc	3218	4	804.50
Sulcia	3978	5	795.60
Acidithiobacillus	3158	4	789.50
Bdellovibrio	2278	3	759.33
Glaciecola	2150	3	716.67
Sphingobium	2129	3	709.67
Methanocaldococcus	3459	5	691.80
Klebsiella	7918	12	659.83
Rhodobacter	3139	5	627.80
Mannheimia	4585	8	573.13
Rhizobium	5011	9	556.78
Acholeplasma	1526	3	508.67
Desulfurococcus	1497	3	499.00
Geobacillus	5271	11	479.18
Arthrobacter	2775	6	462.50
Portiera	2291	5	458.20
Hyphomicrobium	1779	4	444.75
Alteromonas	5742	14	410.14
Blochmannia	1625	4	406.25
Methanobacterium	1194	3	398.00
Yersinia	7235	19	380.79
Rhodopseudomonas	2661	7	380.14
Porphyromonas	1516	4	379.00
Methanocella	1105	3	368.33
Nitrosomonas	1435	4	358.75
Methanobrevibacter	1067	3	355.67
Rhodospirillum	1410	4	352.50
Leuconostoc	2725	8	340.63
Legionella	4291	13	330.08
Lactococcus	4243	13	326.38
Brucella	6507	20	325.35
Acinetobacter	5990	19	315.26
Methylobacterium	2433	8	304.13
Caldicellulosiruptor	2366	8	295.75
Thermoanaerobacter	2274	8	284.25
Enterobacter	2957	11	268.82
Listeria	9012	34	265.06
Flavobacterium	1303	5	260.60
Paenibacillus	2784	11	253.09
Bartonella	2154	9	239.33
Neisseria	4303	18	239.06
Methanosaeta	711	3	237.00
Enterococcus	2532	13	194.77
Thioalkalivibrio	558	3	186.00
Desulfotomaculum	1022	6	170.33
Bradyrhizobium	825	5	165.00
Cyanothece	833	6	138.83
Prochlorococcus	1564	12	130.33

Genus	Number of distinct core *k*-mers	Number of isolates	Number of core *k*-mers per isolate, *K*
Vibrio	3081	24	128.38
Geobacter	1138	9	126.44
Gardnerella	368	3	122.67
Tremblaya	356	3	118.67
Shewanella	2846	24	118.58
Serratia	1275	11	115.91
Deinococcus	792	7	113.14
Prevotella	674	6	112.33
Escherichia	5893	64	92.08
Carsonella	572	7	81.71
Buchnera	993	13	76.38
Propionibacterium	718	14	51.29
Salmonella	2254	46	49.00
Frankia	178	5	35.60
Brachyspira	242	7	34.57
Thermus	236	7	33.71
Staphylococcus	2002	60	33.37
Ehrlichia	187	6	31.17
Pyrococcus	201	7	28.71
Spiroplasma	129	5	25.80
Francisella	383	19	20.16
Desulfovibrio	265	14	18.93
Treponema	208	17	12.24
Corynebacterium	605	51	11.86
Pyrobaculum	75	7	10.71
Lactobacillus	515	57	9.04
Leptospira	60	7	8.57
Archaeoglobus	32	4	8.00
Wolbachia	45	7	6.43
Ruminococcus	23	4	5.75
Anaplasma	45	9	5.00
Ralstonia	53	11	4.82
Chlamydia	391	86	4.55
Xanthomonas	66	15	4.40
Zymomonas	26	6	4.33
Bordetella	17	10	1.70
Sulfolobus	20	17	1.18
Thermococcus	8	9	0.89
Streptomyces	4	19	0.21
Streptococcus	1	123	0.01
