# Supplementary material for: k-mer Similarity, Networks of Microbial Genomes, and Taxonomic Rank
Source: mSystems. 2018 Nov 20;3(6):e00257-18. doi: 10.1128/mSystems.00257-18 (PMC6247013; doi:10.1128/mSystems.00257-18)
Supplement: TABLE S3 [file sys006182296st3.pdf]

Table S3. Number of core *k*-mers in 112 genera of prokaryotes, based on their annotated function in COG functional categories.

Phylum	Genus	No. isolates	COG functional category																							
			A	B	C	D	E	F	G	H	I	J	K	L	M	N	O	P	Q	R	S	T	U	V	W	Y	Z
Actinobacteria	*Clavibacter*	150	2	0	46	11	94	40	96	52	34	39	41	54	51	1	40	77	24	123	102	34	21	13	1	0	0
Actinobacteria	*Nocardia*	115	1	0	69	11	87	29	50	54	33	45	30	43	36	0	35	41	19	78	50	22	15	8	0	0	0
Actinobacteria	*Actinoplanes*	114	0	0	45	8	38	20	37	22	10	22	22	26	17	0	19	28	6	38	19	20	7	5	0	0	0
Actinobacteria	*Amycolatopsis*	102	1	0	91	13	124	49	94	66	40	38	58	61	59	1	51	74	35	157	110	36	15	15	0	0	0
Actinobacteria	*Gordonia*	61	0	0	36	7	49	13	28	31	27	29	17	24	19	0	26	23	9	35	26	11	5	5	0	0	0
Actinobacteria	*Rhodococcus*	35	0	0	10	3	9	3	6	1	7	6	6	6	4	0	7	2	3	4	1	3	1	0	0	0	0
Actinobacteria	*Frankia*	33	0	0	3	1	1	1	6	1	4	2	3	3	5	0	1	2	4	5	4	4	1	2	0	0	0
Actinobacteria	*Arthrobacter*	25	0	0	6	0	9	0	4	2	1	6	1	1	0	0	3	8	1	2	1	1	0	2	0	0	0
Actinobacteria	*Streptomyces*	16	0	0	0	0	7	0	1	1	0	0	0	0	0	0	0	6	0	0	0	0	0	3	0	0	0
Actinobacteria	*Gardnerella*	2	0	0	0	0	0	0	0	0	0	1	0	0	0	0	0	1	1	1	0	0	0	0	0	0	0
Alphaproteobacteria	*Azospirillum*	107	0	0	68	15	76	12	47	28	20	17	18	38	42	12	33	45	12	54	49	52	20	14	0	0	0
Alphaproteobacteria	*Acetobacter*	82	1	0	106	20	135	52	73	98	40	54	44	79	88	1	77	81	20	143	157	30	29	16	1	0	0
Alphaproteobacteria	*Phaeobacter*	82	0	0	10	3	44	3	10	15	10	7	15	20	8	8	13	16	4	15	7	7	5	2	0	0	0
Alphaproteobacteria	*Caulobacter*	73	1	0	36	6	38	6	19	21	25	21	10	20	22	4	22	19	14	31	28	16	16	6	0	0	0
Alphaproteobacteria	*Mesorhizobium*	71	0	0	14	2	30	4	14	14	5	5	9	13	12	0	12	23	6	30	13	11	2	6	0	0	0
Alphaproteobacteria	*Sinorhizobium*	64	0	0	53	5	67	16	45	27	17	19	17	18	12	8	24	34	7	66	36	18	19	7	0	0	0
Alphaproteobacteria	*Bradyrhizobium*	62	0	0	2	0	13	0	7	5	1	1	2	12	4	0	7	17	1	17	6	15	2	6	0	0	0
Alphaproteobacteria	*Oligotropha*	57	0	0	4	0	14	1	9	3	1	3	6	20	7	0	4	22	2	16	9	11	3	5	0	0	0
Alphaproteobacteria	*Rhodobacter*	55	0	0	32	4	27	13	26	16	11	7	6	14	12	1	10	18	4	15	7	2	4	5	0	0	0
Alphaproteobacteria	*Sphingobium*	50	0	0	22	2	13	4	6	8	12	8	1	7	5	0	22	8	1	7	5	9	6	3	0	0	0
Alphaproteobacteria	*Rhodopseudomonas*	43	0	0	2	1	8	1	1	3	2	2	0	7	4	0	4	8	1	11	5	7	2	5	0	0	0
Alphaproteobacteria	*Rhizobium*	39	0	0	11	2	13	1	4	7	1	4	2	4	0	0	8	11	1	9	0	4	0	8	0	0	0
Alphaproteobacteria	*Methylobacterium*	33	0	0	12	2	5	2	6	3	0	3	0	1	7	0	5	4	1	7	4	9	2	2	0	0	0
Alphaproteobacteria	*Agrobacterium*	30	0	0	17	1	15	4	3	4	1	4	1	7	2	0	5	11	1	9	1	3	3	5	0	0	0
Alphaproteobacteria	*Rhodospirillum*	29	0	0	3	2	6	5	1	1	4	0	0	2	1	0	0	9	0	2	1	5	0	2	0	0	0
Alphaproteobacteria	*Brucella*	26	0	0	6	0	10	2	14	6	5	3	7	12	6	0	6	11	0	15	9	3	1	5	0	0	0
Alphaproteobacteria	*Gluconacetobacter*	21	0	0	16	1	8	3	12	11	7	7	8	9	7	0	5	11	2	11	8	3	8	3	0	0	0
Alphaproteobacteria	*Hyphomicrobium*	6	0	0	4	0	1	1	1	0	0	1	0	0	0	0	0	0	0	0	0	0	1	0	0	0	0
Alphaproteobacteria	*Ehrlichia*	2	0	0	1	0	1	0	2	4	1	1	1	0	0	0	2	1	0	0	1	0	2	0	0	0	0
Alphaproteobacteria	*Wolbachia*	2	0	0	0	0	1	1	0	0	1	0	0	0	0	0	0	0	0	0	0	0	0	0	0	0	0
Alphaproteobacteria	*Bartonella*	1	0	0	0	0	1	0	0	0	0	0	0	0	0	0	0	0	0	0	0	0	0	0	0	0	0
Alphaproteobacteria	*Zymomonas*	1	0	0	0	0	0	0	0	0	1	0	0	0	0	0	0	0	0	0	0	0	0	0	0	0	0
Bacteroidetes	*Porphyromonas*	6	0	0	0	0	1	1	0	0	0	1	1	0	0	0	4	0	0	0	2	0	0	0	0	0	0
Bacteroidetes	*Flavobacterium*	2	0	0	0	0	0	0	0	0	0	0	0	0	0	0	0	0	2	0	0	0	0	0	0	0	0
Bacteroidetes	*Riemerella*	1	0	0	0	0	0	0	0	0	0	0	1	0	0	0	0	0	0	0	0	0	0	0	0	0	0
Betaproteobacteria	*Variovorax*	184	1	0	126	20	163	48	109	78	52	72	54	82	92	5	73	112	41	230	204	59	50	19	0	0	0

Phylum	Genus	No. isolates	COG functional category																								
			A	B	C	D	E	F	G	H	I	J	K	L	M	N	O	P	Q	R	S	T	U	V	W	Y	Z
Betaproteobacteria	*Achromobacter*	115	0	0	58	6	81	22	42	34	35	30	35	30	52	20	48	62	28	100	97	27	33	13	0	0	0
Betaproteobacteria	*Cupriavidus*	110	1	0	126	16	130	44	53	77	41	80	36	56	67	12	57	77	23	134	111	43	41	16	0	0	0
Betaproteobacteria	*Acidovorax*	38	0	0	34	4	40	11	9	13	11	12	7	14	7	5	10	18	8	19	17	14	18	4	0	0	0
Betaproteobacteria	*Neisseria*	25	0	0	2	1	4	0	3	4	1	2	1	14	11	0	3	8	3	8	9	1	4	3	0	0	0
Betaproteobacteria	*Bordetella*	14	0	0	0	0	3	0	1	1	0	1	0	0	0	0	1	6	0	6	0	0	0	1	0	0	0
Betaproteobacteria	*Ralstonia*	6	0	0	0	0	0	0	0	0	0	3	0	0	0	0	0	0	0	0	0	4	0	0	0	0	0
Betaproteobacteria	*Taylorella*	6	0	0	14	2	13	1	3	3	1	8	3	5	7	0	4	8	2	10	13	5	2	2	0	0	0
Betaproteobacteria	*Nitrosomonas*	1	0	0	0	0	0	0	0	0	0	0	1	0	0	0	0	0	0	0	0	0	0	0	0	0	0
Chloroflexi	*Chloroflexus*	12	0	0	35	3	31	4	36	19	10	6	11	17	13	0	16	19	8	38	15	10	7	5	0	0	0
Chloroflexi	*Dehalococcoides*	7	0	1	42	7	63	35	19	40	17	28	24	43	14	0	24	21	5	57	37	9	15	2	0	0	0
Crenarchaeota	*Desulfurococcus*	2	0	0	4	0	7	2	3	1	0	5	4	7	0	0	4	2	0	15	3	2	1	0	0	0	0
Cyanobacteria	*Synechocystis*	223	0	0	67	6	76	29	52	43	25	29	17	34	36	0	33	50	13	63	51	24	16	9	0	0	0
Cyanobacteria	*Anabaena*	8	0	0	14	3	11	3	9	9	2	4	3	5	5	0	3	12	6	17	11	3	1	2	0	0	0
Cyanobacteria	*Nostoc*	6	0	0	3	0	3	1	3	2	1	0	2	1	0	0	0	5	1	5	0	0	0	0	0	0	0
Deinococcus-Thermus	*Meiothermus*	36	0	0	15	4	14	5	10	7	7	6	5	17	3	1	7	18	5	22	14	6	6	5	0	0	0
Deinococcus-Thermus	*Thermus*	13	0	0	2	2	4	0	3	1	1	1	1	3	1	0	0	2	1	2	0	0	0	2	0	0	0
Deltaproteobacteria	*Myxococcus*	89	1	0	45	12	60	31	34	42	35	33	28	61	67	3	43	49	15	105	65	50	56	9	0	0	1
Deltaproteobacteria	*Anaeromyxobacter*	74	0	0	77	14	62	27	44	35	32	29	28	53	49	0	48	44	15	91	58	44	36	10	0	0	0
Deltaproteobacteria	*Bdellovibrio*	3	0	0	9	0	1	0	2	0	0	2	1	4	1	0	0	1	0	3	0	2	0	1	0	0	0
Epsilonproteobacteria	*Arcobacter*	4	0	0	13	0	10	5	3	11	3	9	7	7	4	2	6	2	0	7	5	6	4	0	0	0	0
Euryarchaeota	*Haloarcula*	91	0	2	85	11	112	44	55	91	36	55	45	67	24	5	52	74	18	196	174	35	20	13	0	0	0
Euryarchaeota	*Methanosarcina*	3	0	0	12	0	9	5	3	20	1	9	8	16	2	0	5	2	3	13	15	4	1	1	0	0	0
Euryarchaeota	*Methanobrevibacter*	1	0	0	1	0	0	1	0	3	0	0	0	0	0	0	1	0	0	1	0	0	0	0	0	0	0
Euryarchaeota	*Methanocaldococcus*	1	0	0	0	0	0	0	0	0	0	0	0	0	0	0	1	0	0	0	0	0	0	0	0	0	0
Euryarchaeota	*Methanocella*	1	0	0	4	0	2	1	1	0	1	0	1	0	0	0	0	1	0	1	0	0	0	0	0	0	0
Euryarchaeota	*Methanosaeta*	1	0	0	2	0	0	0	0	0	1	0	0	0	0	0	1	0	0	0	0	0	0	0	0	0	0
Euryarchaeota	*Pyrococcus*	1	0	0	1	0	0	0	0	0	0	0	0	0	0	0	0	0	0	0	0	0	0	0	0	0	0
Firmicutes	*Thermoanaerobacterium*	15	0	0	31	12	47	18	43	20	8	22	28	43	35	8	16	17	4	60	52	21	20	4	0	0	0
Firmicutes	*Carnobacterium*	13	0	0	2	3	3	2	9	1	1	10	4	3	0	0	4	1	1	4	3	0	1	0	0	0	0
Firmicutes	*Roseburia*	13	0	0	9	1	21	8	33	2	8	8	7	16	8	6	11	9	0	16	10	12	4	5	0	0	0
Firmicutes	*Desulfitobacterium*	8	0	0	38	4	25	9	8	5	6	19	15	13	15	4	7	11	2	27	26	14	8	2	0	0	0
Firmicutes	*Pediococcus*	8	0	0	2	0	4	1	9	0	3	4	4	4	2	0	2	2	0	6	4	1	1	1	0	0	0
Firmicutes	*Lactococcus*	7	0	0	5	0	0	1	8	0	1	5	3	2	1	0	1	1	0	2	1	0	2	0	0	0	0
Firmicutes	*Desulfosporosinus*	6	0	0	0	0	0	1	0	0	0	1	0	0	1	0	1	0	0	0	2	0	0	0	0	0	0
Firmicutes	*Geobacillus*	4	0	0	2	1	3	0	0	2	0	1	2	1	3	0	0	0	0	0	1	1	1	0	0	0	0
Firmicutes	*Caldicellulosiruptor*	2	0	0	0	0	2	0	0	0	0	0	2	8	1	0	0	0	0	0	1	0	0	1	0	0	0
Firmicutes	*Exiguobacterium*	2	0	0	4	0	0	1	8	0	0	2	0	0	1	0	1	1	0	1	0	0	0	0	0	0	0
Firmicutes	*Thermoanaerobacter*	2	0	0	1	0	1	0	0	1	0	0	0	5	2	0	0	0	0	1	1	0	0	0	0	0	0
Gammaproteobacteria	*Azotobacter*	284	2	0	219	40	225	79	159	136	81	108	90	150	140	26	127	181	62	372	442	105	118	33	0	0	1

Phylum	Genus	No. isolates	COG functional category																								
			A	B	C	D	E	F	G	H	I	J	K	L	M	N	O	P	Q	R	S	T	U	V	W	Y	Z
Gammaproteobacteria	*Stenotrophomonas*	174	2	0	183	36	203	78	109	112	80	96	77	122	145	25	118	138	43	264	283	89	103	26	1	0	0
Gammaproteobacteria	*Edwardsiella*	129	1	0	170	35	171	84	142	118	56	182	68	125	137	19	87	124	22	213	239	69	73	19	0	0	0
Gammaproteobacteria	*Rahnella*	89	1	0	180	42	234	88	206	136	63	200	90	130	145	22	111	167	30	293	296	79	79	25	1	0	0
Gammaproteobacteria	*Shigella*	68	0	0	74	9	101	49	66	62	32	46	43	68	87	1	38	81	25	107	128	49	23	21	1	0	0
Gammaproteobacteria	*Cronobacter*	52	0	0	4	0	7	0	9	3	2	4	3	2	6	1	1	15	3	9	7	6	2	4	0	0	0
Gammaproteobacteria	*Pectobacterium*	50	1	0	49	14	77	31	84	42	19	116	46	35	49	0	40	55	16	95	88	22	25	7	0	0	0
Gammaproteobacteria	*Xylella*	46	1	0	67	17	86	31	43	43	30	37	32	46	66	0	54	40	12	90	84	25	40	15	1	0	0
Gammaproteobacteria	*Aeromonas*	32	0	0	24	3	30	11	23	8	3	6	21	12	12	7	15	29	1	30	14	26	21	7	0	0	0
Gammaproteobacteria	*Erwinia*	32	0	0	22	3	18	7	12	8	0	78	13	17	4	0	6	15	1	4	13	9	12	1	0	0	0
Gammaproteobacteria	*Thioalkalivibrio*	29	0	0	5	2	5	3	4	2	1	5	0	2	2	0	1	5	2	1	1	0	2	2	0	0	1
Gammaproteobacteria	*Pasteurella*	28	0	0	30	6	20	7	31	8	5	35	10	7	10	0	7	14	4	33	23	6	6	1	1	0	0
Gammaproteobacteria	*Coxiella*	20	0	0	4	0	13	4	8	7	6	6	2	19	8	0	4	8	1	15	15	0	9	6	0	0	0
Gammaproteobacteria	*Klebsiella*	20	0	0	3	0	1	1	5	1	0	5	1	0	2	0	2	2	0	5	0	2	0	0	0	0	0
Gammaproteobacteria	*Aggregatibacter*	17	0	0	21	8	22	7	14	16	4	13	6	20	21	0	8	9	1	25	15	3	14	3	0	0	0
Gammaproteobacteria	*Dickeya*	11	0	0	1	0	2	2	3	0	0	6	0	0	0	0	0	2	0	0	3	3	0	0	0	0	0
Gammaproteobacteria	*Alteromonas*	10	0	0	27	3	8	7	1	4	5	24	8	8	1	0	9	0	0	17	8	4	4	0	0	0	0
Gammaproteobacteria	*Marinobacter*	10	0	0	9	1	6	3	2	4	3	7	3	4	2	0	2	3	1	6	0	2	3	0	0	0	0
Gammaproteobacteria	*Pseudoalteromonas*	10	0	0	5	0	0	0	0	1	0	10	0	0	0	0	0	0	0	0	1	1	0	0	0	0	0
Gammaproteobacteria	*Salmonella*	10	0	0	0	1	1	1	2	2	0	3	1	2	0	0	3	0	0	1	0	1	0	2	0	0	0
Gammaproteobacteria	*Yersinia*	10	0	0	0	0	1	1	2	1	0	2	1	0	0	0	0	2	0	1	1	2	0	0	0	0	0
Gammaproteobacteria	*Psychrobacter*	7	0	0	11	1	4	4	1	4	3	6	4	0	2	0	5	1	1	0	3	1	0	0	0	0	0
Gammaproteobacteria	*Actinobacillus*	6	0	0	3	0	6	1	2	1	2	2	1	3	0	0	2	0	0	2	0	0	0	0	0	0	0
Gammaproteobacteria	*Glaciecola*	6	0	0	2	0	0	0	0	1	1	2	1	0	0	0	0	0	0	0	0	0	0	0	0	0	0
Gammaproteobacteria	*Mannheimia*	6	0	0	0	0	0	2	0	0	0	5	0	0	1	0	0	0	0	0	0	1	0	0	0	0	0
Gammaproteobacteria	*Nitrosococcus*	6	0	0	6	2	4	2	7	6	0	3	2	11	7	0	4	6	0	3	2	1	3	0	0	0	0
Gammaproteobacteria	*Pantoea*	6	0	0	0	0	0	0	0	0	0	4	0	0	0	0	0	0	0	1	0	1	0	0	0	0	0
Gammaproteobacteria	*Xanthomonas*	6	0	0	0	0	0	0	0	0	0	2	0	0	0	0	0	0	0	1	0	0	4	0	0	0	0
Gammaproteobacteria	*Marinomonas*	3	0	0	6	0	1	0	1	1	1	0	0	1	0	0	1	2	0	1	0	1	2	0	0	0	0
Gammaproteobacteria	*Acidithiobacillus*	2	0	0	4	1	3	3	1	0	1	3	3	6	1	0	0	2	0	1	1	2	1	0	0	0	0
Gammaproteobacteria	*Enterobacter*	2	0	0	0	0	0	0	0	0	0	2	0	0	0	0	0	0	0	0	0	0	0	0	0	0	0
Gammaproteobacteria	*Escherichia*	2	0	0	0	0	0	0	0	0	0	2	0	0	0	0	0	0	0	0	0	0	0	0	0	0	0
Gammaproteobacteria	*Francisella*	1	0	0	0	0	2	0	1	2	0	0	0	2	0	0	1	0	0	1	0	0	0	0	0	0	0
Gammaproteobacteria	*Serratia*	1	0	0	0	0	0	0	0	0	0	0	0	0	0	0	1	0	0	0	0	0	0	0	0	0	0
Gammaproteobacteria	*Shewanella*	1	0	0	3	0	0	0	0	0	0	0	0	0	0	0	0	0	0	0	0	0	0	0	0	0	0
Spirochaetes	*Brachyspira*	2	0	0	0	0	0	0	0	0	0	0	0	0	0	0	0	0	0	1	0	1	2	0	0	0	0
Tenericutes	*Ureaplasma*	34	0	0	17	7	21	19	20	12	8	27	15	44	9	0	18	23	1	40	28	5	11	7	0	0	0
Thaumarchaeota	*Nitrosopumilus*	2	0	1	33	3	24	10	9	27	6	12	11	17	5	0	15	12	2	21	20	1	5	2	0	0	0
