# Supplementary material for: k-mer Similarity, Networks of Microbial Genomes, and Taxonomic Rank
Source: mSystems. 2018 Nov 20;3(6):e00257-18. doi: 10.1128/mSystems.00257-18 (PMC6247013; doi:10.1128/mSystems.00257-18)
Supplement: TABLE S4 [file sys006182296st4.pdf]

Table S4. Comparison of annotated core *k*-mers in each phylum against all annotated core *k*-mers, based on COG functional categories. Adjusted *p*-values are shown for each comparison, those ≤ 0.05 (highlighted in bold-face and red shade) are considered statistically significant.

Phylum	A	B	C	D	E	F	G	H	I	J	K	L	M	N	O	P	Q	R	S	T	U	V	W	Y	Z
Actinobacteria	0.282	1.000	0.564	1.000	**0.041**	0.216	**5.13E-5**	0.340	**0.011**	**0.046**	**0.019**	0.687	0.340	**06.85E-8**	1.000	0.687	**0.001**	1.000	**6.05E-4**	0.431	**3.51E-9**	1.000	0.800	1.000	1.000
Alphaproteobacteria	1.000	1.000	0.057	1.000	**4.22E-5**	**0.030**	1.000	0.154	0.154	**7.04E-10**	0.084	0.566	0.864	0.438	**0.010**	**0.004**	0.753	**0.010**	**1.36E-8**	**0.011**	0.072	**1.76E-5**	1.000	1.000	1.000
Bacteroidetes	1.000	1.000	1.000	1.000	1.000	1.000	1.000	1.000	1.000	1.000	0.671	1.000	1.000	1.000	0.060	1.000	0.269	1.000	1.000	1.000	1.000	1.000	1.000	1.000	1.000
Betaproteobacteria	1.000	1.000	0.665	0.289	0.208	0.208	**0.006**	0.390	0.665	0.192	0.208	**0.009**	0.665	**0.009**	1.000	0.374	**0.006**	0.208	0.208	1.000	0.509	1.000	1.000	1.000	1.000
Chloroflexi	1.000	0.245	0.277	1.000	0.135	0.135	0.896	0.109	0.896	0.245	0.531	0.165	0.109	0.109	0.896	0.245	1.000	0.826	**0.019**	0.174	0.847	0.531	1.000	1.000	1.000
Crenarchaeota	1.000	1.000	1.000	1.000	1.000	1.000	1.000	1.000	1.000	1.000	1.000	0.705	0.705	1.000	1.000	1.000	1.000	0.068	1.000	1.000	1.000	1.000	1.000	1.000	1.000
Cyanobacteria	1.000	1.000	0.222	0.914	0.323	0.640	0.323	0.323	0.955	0.268	0.323	0.602	1.000	0.222	1.000	0.222	0.323	0.914	0.222	1.000	0.291	1.000	1.000	1.000	1.000
Deinococcus-Thermus	1.000	1.000	1.000	0.345	1.000	1.000	1.000	1.000	1.000	0.687	1.000	0.271	0.318	1.000	1.000	0.345	0.686	1.000	0.686	1.000	1.000	0.271	1.000	1.000	1.000
Deltaproteobacteria	0.873	1.000	0.934	0.873	**0.013**	1.000	**0.020**	0.382	0.089	**0.007**	0.873	0.057	**0.008**	**0.046**	0.277	0.438	1.000	0.438	**0.004**	**1.06E-4**	**1.14E-5**	0.873	1.000	1.000	0.288
Epsilonproteobacteria	1.000	1.000	0.545	0.732	1.000	0.732	0.578	0.545	1.000	0.578	0.545	0.931	0.931	0.545	0.815	0.545	0.641	0.578	0.545	0.641	0.901	0.732	1.000	1.000	1.000
Euryarchaeota	1.000	**0.032**	0.792	0.187	0.792	0.792	**0.004**	**1.67E-5**	0.792	0.414	0.792	0.792	**4.88E-9**	0.709	0.827	0.699	0.792	**7.77E-6**	**1.07E-5**	0.337	**7.49E-4**	0.705	1.000	1.000	1.000
Firmicutes	1.000	1.000	1.000	0.549	1.000	1.000	**2.38E-5**	**1.01E-4**	0.339	0.549	**0.004**	**0.003**	0.549	**0.002**	0.339	**3.22E-4**	**0.003**	0.549	0.482	0.549	1.000	0.944	1.000	1.000	1.000
Gammaproteobacteria	0.914	0.210	**0.029**	0.210	**2.21E-6**	1.000	0.914	**0.018**	**6.47E-4**	**5.50E-15**	0.462	**0.021**	**0.002**	0.177	0.072	0.210	**0.009**	0.210	**6.59E-15**	0.210	**4.31E-5**	**0.029**	0.210	1.000	0.690
Spirochaetes	1.000	1.000	1.000	1.000	1.000	1.000	1.000	1.000	1.000	1.000	1.000	1.000	1.000	1.000	1.000	1.000	1.000	1.000	1.000	1.000	0.146	1.000	1.000	1.000	1.000
Tenericutes	1.000	1.000	0.180	0.550	0.182	0.180	1.000	0.493	0.903	0.182	0.728	**7.55E-6**	0.180	0.573	0.842	0.903	0.180	0.842	0.818	0.180	1.000	0.550	1.000	1.000	1.000
Thaumarchaeota	1.000	0.136	**0.022**	1.000	0.963	0.839	0.457	**0.005**	1.000	1.000	0.839	0.839	0.136	0.839	0.763	0.839	0.839	0.839	0.910	**0.022**	0.839	1.000	1.000	1.000	1.000
