# Supplementary material for: k-mer Similarity, Networks of Microbial Genomes, and Taxonomic Rank
Source: mSystems. 2018 Nov 20;3(6):e00257-18. doi: 10.1128/mSystems.00257-18 (PMC6247013; doi:10.1128/mSystems.00257-18)
Supplement: FIG S4 [file sys006182296sf4.pdf]

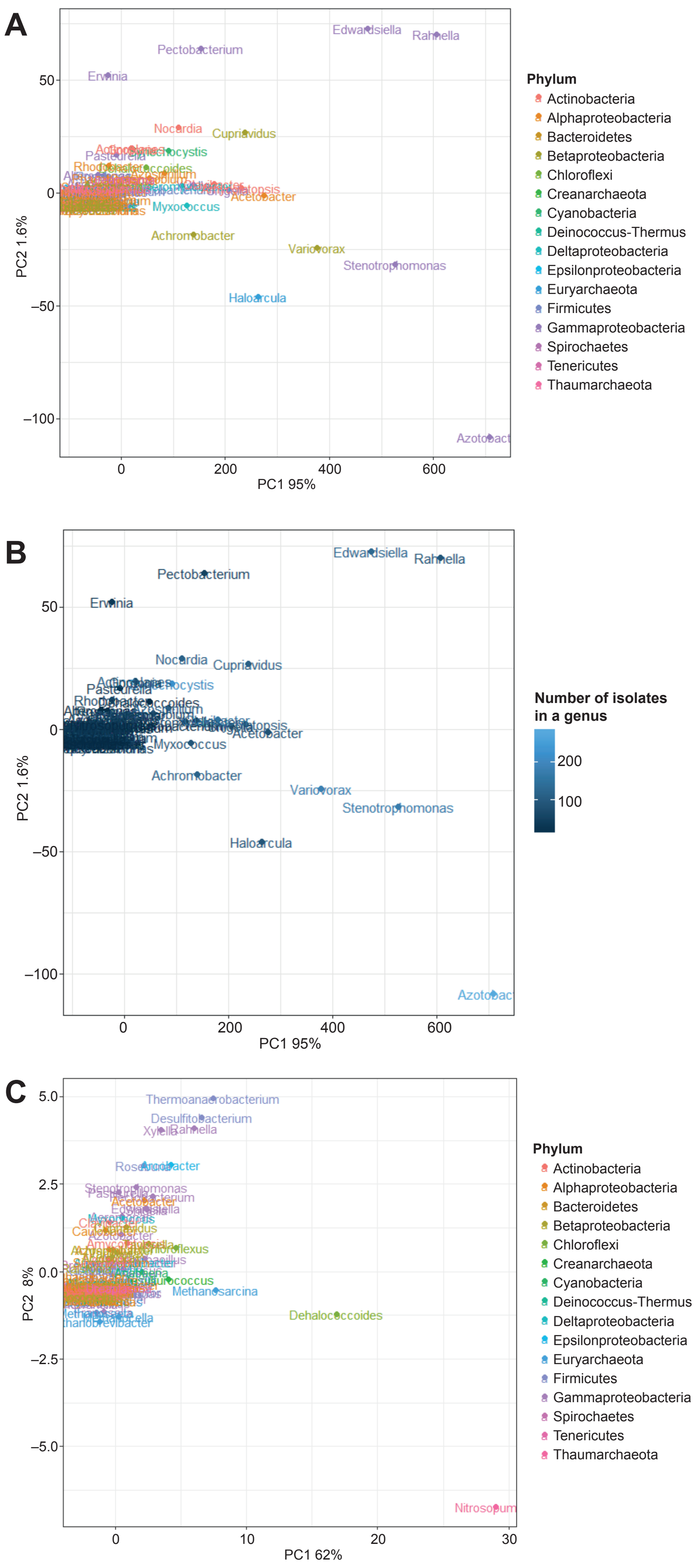

Figure S4. Principal component analysis (PCA) of core *k*-mers and their annotated COG categories, based on core *k*-mers in each (A) phylum and (B) genus. The PCA of core *k*-mers in each phylum performed on the normalised counts of COG categories in centred scale is shown in (C).
