# Supplementary material for: k-mer Similarity, Networks of Microbial Genomes, and Taxonomic Rank
Source: mSystems. 2018 Nov 20;3(6):e00257-18. doi: 10.1128/mSystems.00257-18 (PMC6247013; doi:10.1128/mSystems.00257-18)
Supplement: TEXT S1 [file sys006182296s1.pdf]

Text S1

Computational scalability and runtime of AF phylogenomics

In this study, we used a modified version of our own implementation of the D_2 statistics (1). This newer version was used to compute the D_2^S distance of two genomes at a time. Each pairwise distance can be computed independently, so we ran thousands of parallel jobs for each pairwise comparison for our different AF networks across a high-performance distributed-memory computing cluster. Although the time can vary depending on genome size, on average, it takes about five seconds to compute the D_2^S distance between two microbial genomes. The principal advantage of this approach is that it is not limited by memory, as each job requires only a few hundred MB. Although visualization of the network using the D3 library is scalable to large data, it can take a few minutes for the force-directed algorithm to provide an optimal layout for a densely connected network.

Extraction of the core *k*-mers required less than an hour for our dataset of 2783 microbial genomes. Mapping the core *k*-mers of 1475 genomes to our SQL database on a SSD hard drive took less than one hour. Assessment of computational scalability was carried out using a high-performance distributed-memory computing cluster based on Intel Xeon Haswell (3.1 GHz) cores. Comparative runtime analysis of alignment-free methods was made on Intel Xeon Haswell E5-2667 v3 cores rated at 3.1 GHz, using a single processor and one thread.

A major advantage of AF approaches in general (and this approach in particular) lies in its computational performance in the inference of phylogenetic networks, and the extraction and mapping of core *k*-mers to biological function (1, 2). Because our approach utilises independent pairwise comparisons we can distribute the computation across multiple processors, greatly minimizing problems potentially arising due to demand on memory (1).

Here we inferred 25-mer similarity networks among more than 2700 genomes in a matter of hours. To map core *k*-mers to our database we took advantage of the SQL architecture, indexing and hashing to compare billions of *k*-mers in a few minutes using an SSD hard drive. The database itself could be generated in only a few hours from RefSeq data for more than 4000 microbial isolates.

It would be of great interest to be able to discriminate edges in the AF networks based on the dominant phylogenetic signal observed (e.g. vertical *versus* lateral), or to color them by proportion of vertical or lateral *k*-mers. To visualize large phylogenetic network such as those presented here, the D3 library (and web technology more generally) might not be optimal. Indeed, even with recent improvements of the JavaScript-based application and an optimized library such as D3, it is difficult for web browsers to display large networks in a force-directed layout. An alternative might be to use software designed specifically for visualization of large networks, e.g. Gephi (3), although this would undoubtedly come at the expense of accessibility e.g. through unfamiliarity among users, or loss of cross-browser compatibility. An open-access, publicly available *k*-mer database would be useful for our research community; such a database would require dedicated long-term infrastructure, management and support.

References

1. Bernard G, Chan CX, Ragan MA. 2016. Alignment-free microbial phylogenomics under scenarios of sequence divergence, genome rearrangement and lateral genetic transfer. Sci Rep 6:28970.

2. Greenfield P, Roehm U. 2013. Answering biological questions by querying k-mer databases. Concurrency and Computation: Practice and Experience 25:497-509.

3. Bastian M, Heymann S, Jacomy M. 2009. Gephi: an open source software for exploring and manipulating networks. Proceedings of the Third International Conference on Weblogs and Social Media (ICWSM) 8:361-362.
